# Pressure-Induced Detrapping from Self-Trapped Excitons to Free Excitons toward Enhanced Emission and Piezochromism in Ruddlesden–Popper (110)-Oriented Perovskites

**DOI:** 10.1021/acsami.5c14096

**Published:** 2025-10-09

**Authors:** Mirosław Mączka, Szymon Sobczak, Kinga Roszak, Daniel Linhares Militão Vasconcelos, Filip Dybała, Artur P. Herman, Robert Kudrawiec, Andrzej Katrusiak, Paulo T. C. Freire

**Affiliations:** † Institute of Low Temperature and Structural Research, Polish Academy of Sciences, Okólna 2, 50-422 Wroclaw, Poland; ‡ Department of Materials Chemistry, Faculty of Chemistry, 49562 Adam Mickiewicz University, Uniwersytetu Poznańskiego 8, 61-614 Poznań, Poland; § Faculdade de Educação, Ciências e Letras do Sertão Central, Universidade Estadual do Ceará, 60714-903 Quixadá, CE, Brazil; ∥ Department of Semiconductor Materials Engineering, Faculty of Fundamental Problems of Technology, Wrocław University of Science and Technology, Wybrzeże Wyspiańskiego 27, 50-370 Wrocław, Poland; ⊥ Departamento de Fisica, 28121 Universidade Federal do Ceara, P.O. Box 6030, 60455-970 Fortaleza, Brazil

**Keywords:** perovskite, lead halide, high-pressure, crystal structures, photoluminescence, phase transitions

## Abstract

Two-dimensional (2D) lead halide perovskites are emerging as excellent materials for optoelectronic applications including light-emitting diodes, photovoltaics, and photodetectors, owing to their efficient excitonic emission originating from both self-trapped excitons (STEs) and free excitons (FEs). Recently, many efforts have been focused on enhancing the emission intensity, modulating the dominant emission mechanism, and establishing direct correlations between optical properties and underlying structural motifs. Across the hybrid organic–inorganic layers in 2D perovskites, the nanorange modulation of the density, stiffness, strain, and ionicity can be efficiently tuned by external stimuli, including the substrate–film strain. Here, we report a pressure-induced narrowing of the band gap and an enhancement of STE and FE emission in the topology of the (110)-oriented Ruddlesden–Popper (RP) perovskite ACE_2_PbBr_4_ (ACE = acetamidinium), accompanied by the rare phenomenon of the reversible detrapping process from STEs to FEs under compression. Specifically, the STE-related emission exhibits a 6.4-fold increase of intensity up to 2.56 GPa, while the FE emission dominates under higher pressures, up to 11.11 GPa, causing a pronounced change in the emission color, from orange-yellow to greenish-blue, across the compression range. In situ single-crystal X-ray diffraction and Raman spectroscopy reveal that these emission changes arise from the rarely observed pressure-induced reduction of lead bromide octahedral distortion and confinement in the direction of the corrugated structure and changes in amine-framework interactions, as well as pressure-induced phase transitions (PTs) occurring near 2, 5, 5.9, and 6.7 GPa. These findings elucidate the structure–property relationship in (110)-oriented RP perovskites and underscore the utility of strain engineering for realizing light-emitting materials with tailored and enhanced functionalities.

## Graphical abstract

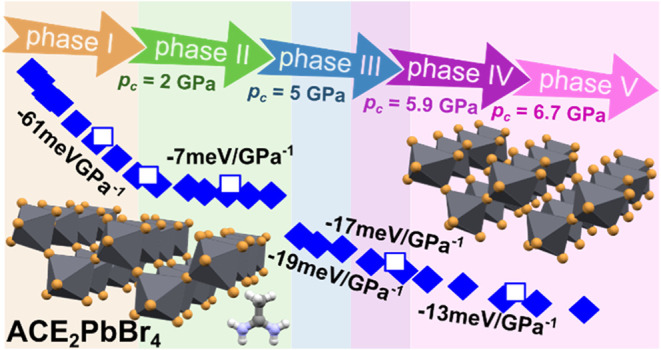

## Introduction

Hybrid organic–inorganic lead halides received enormous interest in recent years as functional materials for various applications including solar cells, photodetectors, and light-emitting devices.^1–4^ One of the very promising groups is lead halides crystallizing in a three-dimensional (3D) perovskite structure of the general formula APbX_3_ (A = small organic cation; X = halide anion).^1,2,5–12^ Unfortunately, 3D lead halide perovskites are scarce due to the subnanometer size of the perovskite voids available for organic cations, and such structures were reported for four small organic cations only.^5–12^ Consequently, the prototypic 3D perovskites are isotropic or gently anisotropic due to symmetry-reducing PTs.^13^ In order to overcome the problem of the small structural diversity of 3D perovskites, lead halides comprising larger organic cations can be designed. This approach led to the discovery of enormous amounts of lower-dimensional hybrid lead halides, but the most important and the largest subgroup consists of 2D analogues of the general formula A′_2_PbX_4_ (RP) and A″PbX_4_ (Dion–Jacobson, DJ), where A’ and A″ denote monovalent and divalent organic cations, respectively.^14–19^ These perovskites are derived from the 3D structure by replacing inorganic layers along crystallographic planes (001), (110), or (111) with the layers composed of organic cations.^14,15^ Due to the presence of isolating organic layers, such systems are natural quantum-well structures exhibiting wider band gaps and larger exciton binding energies compared to the 3D analogues, making 2D analogues very promising for light-emitting applications.^14–16,20,21^ In this respect, emission of the (001)-oriented perovskites is usually related to the radiative recombination of FEs, leading to the narrow emission of high color purity, attractive for fabrication of light-emitting diodes (LEDs).^14–16,21,22^ On the other hand, the presence of corrugated zigzag inorganic planes in (110)-oriented perovskites enhances electron–phonon coupling between the exciton and the inorganic lattice, leading to the formation of STEs. As a result, the photoluminescence (PL) of such systems is usually dominated by a broadband emission related to the recombination of STEs.^14,15,23,24^ This type of emission can be used for designing of color displays, lighting, and white-light LEDs.

Optical properties of 2D lead halides can be tuned by changing the chemical composition because the shape, size, and ability to form hydrogen bonds (HBs) of the organic cation decides which type of the layered structure, (001)-, (110)-, or (111)-oriented, will be realized and how large will be the octahedral distortion. The majority of 2D perovskites reported in the literature crystallize with the (001)-oriented structure and the increase of the octahedral distortion widens the band gap, leading to a blue shift of the FE emission.^14,15,18,25,26^ (110)- or (111)-oriented analogues are less common and their properties are less explored, but literature data show that in these compounds, the Stokes shift and width of the STE bands increase with increasing distortion of the inorganic layers.^14,15,23,25^

Optical properties can also be controlled by the change of temperature, as it affects key parameters such as exciton binding energy, exciton–phonon coupling, dynamics of organic cations, and structural distortion of the perovskite layers.^14,15,18,22,25,26^ Another thermodynamic parameter that allows the tuning of the physicochemical properties of materials is pressure. However, pressure induces significantly stronger changes in the perovskite structure than changes of temperature. As a result, the application of pressure leads to a more pronounced modulation of optoelectronic properties and may lead to the onset of new high-pressure phases with enhanced or unusual properties, such as an enhanced PL intensity or negative compressibility along one or two crystallographic directions.^27–30^ Thus far, high-pressure studies have focused predominantly on (001)-oriented perovskites.^27–32^ These investigations revealed that in the low-pressure regime (up to 5 GPa), compression usually leads to a red shift of the FE emission, but this trend often reverses to a blue shift at higher pressures due to increased deformations of the inorganic layers.^30,33,34^ In certain cases, octahedral distortion and enhanced electron–phonon interactions at high pressure are sufficient to trap the excitons, leading to the appearance of a broadband STE-related PL.^27,35^ Very recently, a very rare phenomenon of pressure-induced detrapping from STEs to FEs was realized in RP *R*- and *S*-[4MeOPEA]_2_PbBr_4_ (4MeOPEA = 4-methoxy-***α***-methylbenzylammonium), leading to a substantial tunability of the emission color.^36^ This transfer was attributed to a decreased Pb–X bond length, a decreased bond-length distortion (Δ*d*), and a suppressed magnitude of molecular motions on compression, which together can result in reduced electron–phonon coupling.^36^ Literature data also indicate that the behavior of RP lead halide perovskites comprising small organic cations may significantly differ from the behavior of the analogues with large organic cations. Notably, we previously demonstrated that the compact size of molecular species may allow them to penetrate voids within the perovskite layers, imparting unusual optical and mechanical properties.^30^

In contrast to (001)-oriented perovskites, the pressure-induced structural and optical responses of (110)-oriented analogues remain almost unexplored. To the best of our knowledge, high-pressure investigations were reported for two such systems, i.e., DJ (API)­PbBr_4_ (API = *N*-(3-aminopropyl)­imidazole) and the alternative cation in the interlayer space (ACI) perovskite IMMHyPbBr_4_ (IM = imidazolium, MHy = methylhydrazinium).^37,38^ IMMHyPbBr_4_ showed stable broadband STE-related emission up to 10.1 GPa, whereas (API)­PbBr_4_ exhibited a distinct transition from STE- to FE-dominated PL with increasing pressure, accompanied by approximately a 5000-fold enhancement in UV light responsivity, making this perovskite very promising for UV photodetection applications. Interestingly, in (API)­PbBr_4_, the transition from STEs to FEs (indicative of weakened electron–phonon coupling) occurred despite increasing octahedral distortion (Δ*d*) upon compression. This counterintuitive behavior was attributed to reduced confinement along the direction of the corrugated framework under pressure, which increases electronic dimensionality and thereby suppresses STE formation.^38^

Inspired by the observation that the light-emitting properties of 2D perovskites can be strongly tuned and enhanced under compression, particularly in systems containing small organic cations, and recognizing that the effect of pressure on the optical properties of (110)-oriented RP analogues remains unexplored, we undertook a comprehensive investigation of lead bromide comprising a small acetamidinium cation (ACE_2_PbBr_4_, ACE = acetamidinium) as a representative example of this class of lead halide perovskites. We used complementary in situ X-ray diffraction, Raman spectroscopy, absorption, and PL measurements as a function of pressure, along with temperature-dependent optical studies. We observed the very rarely reported simultaneous pressure-induced enhancement of both broadband and narrowband emissions up to 2.56 GPa, followed by the suppression of STE emission associated with further enhancement, abrupt weakening, and subtle modulation of the FE emission intensity up to ∼5 GPa, in the 5–6 GPa range and above 6 GPa, respectively. By employing high-pressure single-crystal X-ray diffraction methods and Raman spectroscopy, we attributed the PL and transmission changes at 2, 5, 5.9, and 6.7 GPa to pressure-induced PTs. We have also correlated the observed and rarely reported transfer from the STEs to FEs with changes in structural and phonon characteristics. This work provides a deep insight into the interplay between the structure, phonons, and PL properties in (110)-oriented perovskites under high pressure.

## Experimental Section

### Synthesis

All reagents were commercially purchased from Sigma-Aldrich and used without further purification (PbBr_2_ 99%, HBr 48% in H_2_O, acetamidine hydrochloride).

In order to grow single crystals of ACE_2_PbBr_4_, 2 mmol of PbBr_2_ and 10 mmol of 2 acetamidine hydrochloride were dissolved in HBr at 50 °C under stirring for 5 h. Then, the temperature was lowered to 35 °C, and the vial was left undisturbed. Transparent colorless crystals, which grew at the bottom of the vial, were separated from the liquid after 1 day and dried at RT.

### High-Pressure Single-Crystal X-ray Diffraction

High-pressure single-crystal X-ray diffraction (SCXRD) experiments were performed using a modified Merrill–Bassett diamond anvil cell (DAC).^39^ The DAC was equipped with type Ia diamonds featuring 0.8 mm culet diameters, mounted on stainless steel backing plates with conical openings to optimize the angular access formed by drilling a 0.45 mm hole into a preindented Inconel gasket (final thickness: 0.2 mm) using an electrospark discharge method. A small ruby chip was placed alongside the sample to determine the pressure *in situ* via the ruby R1-line fluorescence method,^40^ ensuring an accuracy of ±0.02 GPa. To maintain hydrostatic conditions, Daphne 7575 oil was used as the pressure-transmitting medium for measurements of up to 4.2 GPa. The measurement performed at 4.64 GPa in Daphne oil was significantly affected by quasi-hydrostatic conditions; therefore, only unit-cell parameters are reported. For higher-pressure experiments, a 1:1 volumetric mixture of *n*-pentane and *iso*-pentane was employed, which remains hydrostatic up to 7.4 GPa.^41^ The pressure was determined before and after each diffraction experiment, and all measurements were conducted at room temperature (RT ∼298 K). The high-pressure SCXRD data were collected using an Oxford Diffraction Xcalibur diffractometer equipped with a CCD detector and Mo Kα radiation (λ = 0.71073 Å). The DAC was precisely centered on the goniometer using the gasket shadowing method.^42^ Diffraction images were recorded using φ = 0° mode and ω scans, ensuring maximal reciprocal space coverage and an exposure time of 30 s per frame. Raw diffraction data were processed using CrysAlisPro software.^43^ The DAC absorption, gasket shadowing, and sample absorption were calculated using the Redshabs program.^44^ The crystal structures were solved by direct methods using SHELXS within the Olex^2^ software suite, and full-matrix least-squares refinements on *F* ^2^ were performed with SHELXL.^45–47^ Nonhydrogen atoms were refined anisotropically where data permitted, while hydrogen atoms were placed in calculated positions by using a riding model. Geometric restraints (e.g., ISOR) were applied to lighter atoms where necessary to maintain the refinement stability. The final crystallographic models were evaluated based on residual factors, goodness-of-fit statistics, and structural consistency across the pressure range. Compressibility data and additional structural parameters, atomic coordinates, and refinement details are available in Tables 1 and S1–S6.

**Table 1:** Selected Crystallographic Data of ACE_2_PbBr_4_

phase		I	II	II	III	III	IV	V
pressure (GPa)		1.89	2.10	4.20	5.20	5.70	6.20	6.80
space group		*P*2_1_/*n*	*P*2_1_/*n*	*P*2_1_/*n*	*P*2_1_/*n*	*P*2_1_/*n*	*P*2_1_/*n*	*P*1̅
unit cell	*a* (Å)	12.237(12)	12.220(16)	12.05(3)	11.947(7)	11.859(6)	11.499(5)	11.438(4)
	*b* (Å)	8.9874(13)	8.9082(12)	8.535(4)	8.4621(4)	8.4242(3)	8.376(4)	8.441(6)
	*c* (Å)	11.847(2)	11.8058(10)	11.603(5)	34.5859(11)	34.4721(10)	11.84(5)	11.4999(15)
	α (deg)	90	90	90	90	90	90	90.58(3)
	β (deg)	92.40(4)	92.80(3)	94.69(12)	94.414(11)	94.556(11)	94.15(12)	94.296(18)
	γ (deg)	90	90	90	90	90	90	90.48(4)
volume (Å^3^)		1301.8(13)	1283.7(16)	1189(3)	3486.1(19)	3432.9(19)	1137(5)	1107.1(9)
*Z/Z*′		4/1	4/1	4/1	12/3	12/3	4/1	4/2
*D*_*x*_ (g/cm^3^)		3.291	3.338	3.602	3.687	3.736	3.767	3.870

### Raman Spectroscopy

The high-pressure Raman experiment was conducted using the Jobin Yvon T64000 triple spectrometer, equipped with a CCD detection system cooled with liquid nitrogen and focused with an Olympus 20× objective, with a focal length of 20.5 mm and a numerical aperture (NA) of 0.35. The excitation source used was an Ar–Kr laser with a wavelength of 514.5 nm and an output power of approximately 100 mW. In this experiment, a Panoramix MDAC diamond anvil cell was used with the pressure controlled via a membrane. The gasket used was composed of preindented stainless steel, with an opening of approximately 140 μm in diameter between the 350 μm tables of the diamonds. The sample and a ruby were placed inside the gasket hole along with the pressure-transmitting medium, which consisted of high-purity mineral oil (Nujol) from União Química Farmacêutica Nacional S/A, ensuring the preservation of hydrostaticity. The comparison of the Raman spectra recorded for the mineral oil and ACE_2_PbBr_4_ outside the DAC with the ambient pressure spectra of ACE_2_PbBr_4_ inside the DAC shows that all Raman bands analyzed in this paper originate from ACE_2_PbBr_4_ (Figure S1). The pressure value was determined by measuring the luminescence of the ruby placed inside the pressure chamber.

### Temperature-Dependent PL and Reflectance Measurements

For PL measurements, the sample was excited with a 325 nm line from a HeCd laser (Kimmon IK3501R-G) with a power of 0.25 mW, which when focused on the sample, gives an excitation density of ∼0.3 W/cm^2^. PL spectra were measured using an experimental setup equipped with a Peltier-cooled Avantes CCD spectrometer (AvaSpec-ULS2048X64TEC-EVO) and appropriate optics. For reflectance (R) measurements, the sample was illuminated with white light from a halogen lamp. A 0.5 m monochromator (Zolix Omni-λ series) was used to disperse the reflected light from the sample. The light was detected by a silicon photodiode by using the lock-in technique with an SR830 DSP lock-in amplifier. For PL and R measurements with the temperature, the sample was placed inside a closed-cycle cryostat, which allowed optical measurements from 10 to 350 K.

### High-Pressure Optical Studies

For transmission and PL measurements under hydrostatic pressure, the sample was mounted in the DAC (Diacell CryoDAC-Nitro). To determine the pressure inside the DAC, a ruby sphere was used. The sample and ruby sphere were excited with a 325 nm HeCd laser (Kimmon IK3501R-G). The excitation density was estimated to be ∼1 W/cm^2^ before the DAC. The PL signal was analyzed using a 0.5 m Andor monochromator and a Si CCD camera cooled down to −70 °C by Peltier elements. A halogen lamp was used as a light source for the transmission measurements. The sample was illuminated with full-spectrum white light, and a CCD spectrometer was used to analyze the transmitted light. The light was focused onto the sample in the DAC using a 10× mirror objective with a long working distance. A second identical objective was used to collect the light transmitted through the sample.

## Results and Discussion

### Single-Crystal X-ray Diffraction

At ambient conditions, ACE_2_PbBr_4_ crystallizes in the monoclinic *P*2_1_/*c* space group (phase I), adopting a 2D perovskite structure, where PbBr_6_ octahedra form corrugated 2 × 2 (110)-oriented perovskite layers separated by acetamidinium (ACE^+^) cations (Figure 1).^48^ These cations penetrate the corrugation grooves of the (PbBr_4_)^2–^ *_n_* polyanion and the corner-sharing PbBr_6_ octahedra arrange into a periodic corrugation displaying a considerable strain in Br–Pb–Br angles significantly deviating from the expected 180°, i.e.,166.45° measured along the [210] direction and 162.35° along the [001] direction. These deviations prevent the formation of a perfectly aligned octahedral framework and are a key factor influencing its mechanical properties under compression.

**Figure 1:**
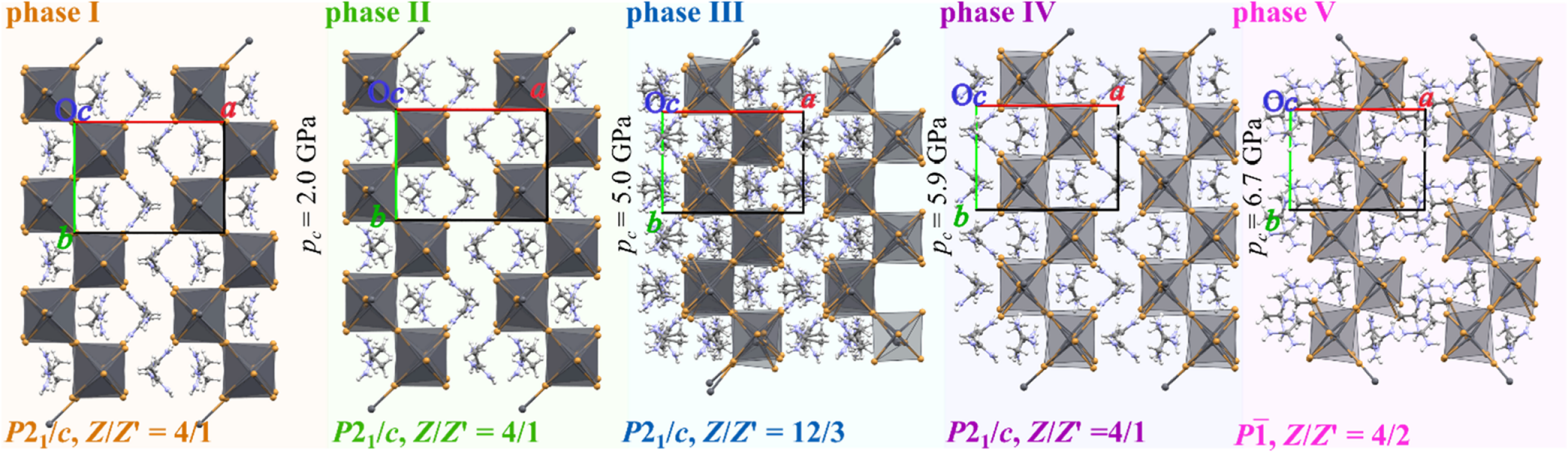
Crystal structures of the ACE_2_PbBr_4_ phases I–V viewed down the *c* axis, illustrating the pressure-induced deformation of corrugated layers. Polyhedral representations highlight the PbBr_6_ octahedra (gray), while organic ACE^+^ cations are shown as ball-and-stick models. Color code of atoms: C, gray; H, white; N, blue for ACE^+^; Br linkers: orange and black faces of PbBr_6_ octahedra.

ACE_2_PbBr_4_ is monotonically compressed to up to 2 GPa. The bulk modulus (*B*_0_), obtained from fitting the second-order Birch–Murnaghan equation of state, calculated with the program PASCal,^49,50^ is *B*_0_ = 14.0 (15) GPa for phase I (Table S1). The compression of this phase is nearly isotropic (Figures 2a and S2), with the compressibility coefficients calculated as β_*x*_ = −(1/*x*)­(∂*x*/∂*p*), where *x* represents the unit-cell parameters, β_a_ = 0.020(2) GPa^–1^, β_b_ = 0.018(9) GPa^–1^, and β_c_ = 0.019(18) GPa^–1^ (Table S3). This behavior contrasts strongly with the anisotropic compression observed in (001)-oriented perovskites, where the highest compressibility occurs perpendicular to the inorganic layers due to much weaker interactions formed between countercations and polyanionic sheets compared to bonds within layers.^27,30,32,36^ In (110)-oriented perovskites, the interlayer spacing is significantly smaller, resulting in denser packing and stronger cation–polyanion interactions. Indeed, previous studies on (110)-oriented (API)­PbBr_4_ reported only a small reduction in interlayer spacing under compression up to 10 GPa, accompanied by a strong deformation of coordination octahedra.^38^ In contrast to (API)­PbBr_4_, where both the bond-length distortion (Δ*d*) and the bond-angle variance (σ^2^) continuously increase across the investigated pressure range,^38^ the structural deformation parameters calculated for PbBr_6_ octahedra of ACE_2_PbBr_4_ (for the calculation equations, see the Supporting Information) behave differently; Δ*d* initially strongly increases and then systematically decreases, whereas σ^2^ significantly increases (Tables S7 and S9 and Figure 2b).

**Figure 2:**
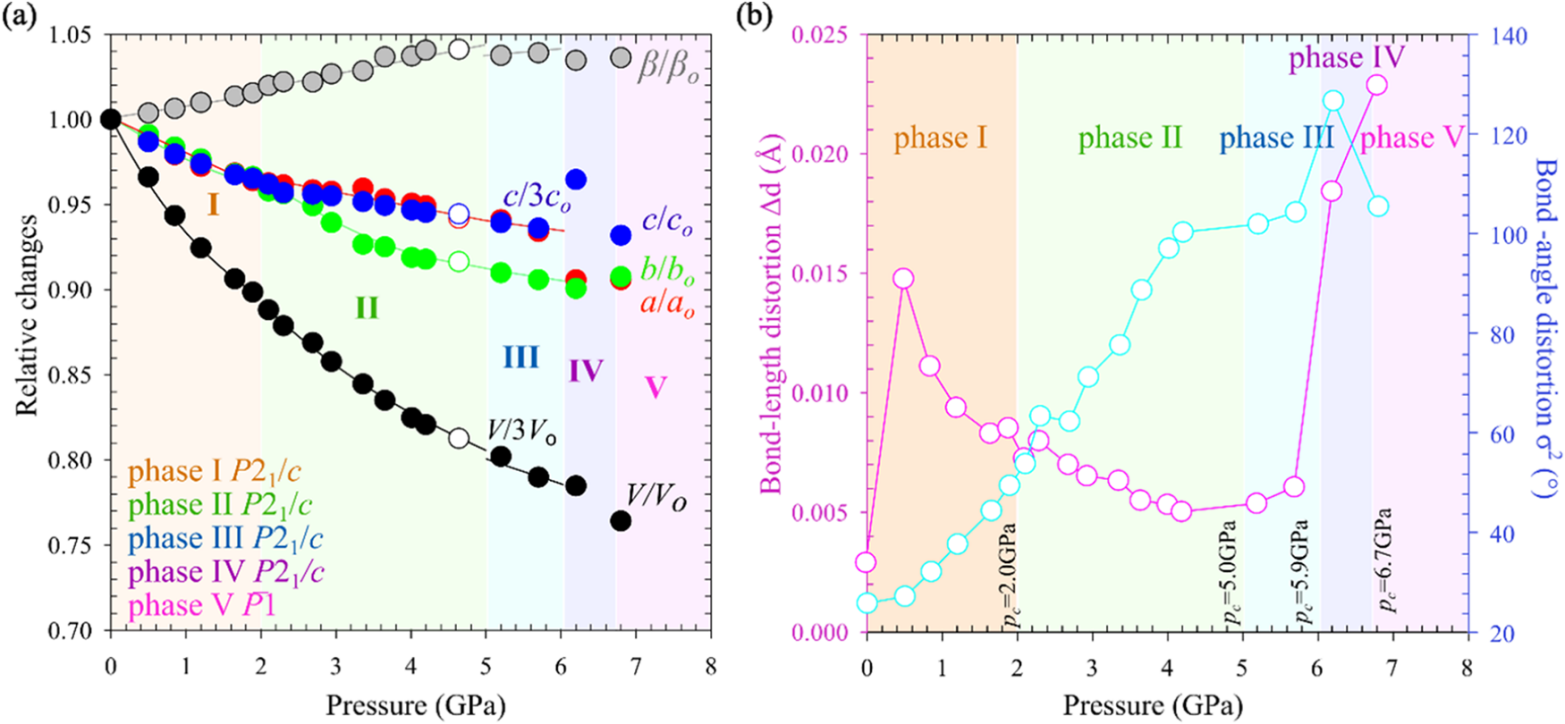
(a) Compression of the unit-cell parameters in ACE_2_PbBr_4_ related to the unit-cell parameters at 0.1 MPa: *a*_0_, *b*_0_, *c*_0_, *V*_0_ and β_o_ and (b) PbBr_6_ octahedra distortion measured by the mean bond-length deviation Δ*d* (plotted in pink) and the bond-angle variance σ^2^ (blue), cf. Tables S7–S10. Shaded backgrounds mark the stability ranges of phases I–V, and critical pressures (*p*_c_) are indicated. The ESDs are smaller than the plotted symbols.

Above 2 GPa, the *b-*axis, running across the folds of the corrugated polyanion (Figure 1), becomes significantly more compressible, whereas the layer-stacking direction *a* and the in-layer direction *c*, running along the folds, become considerably stiffer (Figures 2a and S2), marking the onset of an isostructural PT to phase II, of the same monoclinic space group *P*2_1_/*c*. Consequently, compressibility along the direction *b* exceeds that along the directions *a* and *c*, with the compressibility coefficients as follows: β_a_ = 0.013(5), β_b_ = 0.0185(12), and β_c_ = 0.008(4) TPa^–1^ (Table S3). The bulk modulus of phase II decreases to *B*_0_ = 12.4(8) GPa (Table S2).

The isostructural PT to phase II ‘gently adjusts’ the unit-cell parameters of phase I, with the monoclinic angle β increasing monotonically within phase I, then abruptly but weakly increasing through the critical pressure, and then continuing to increase in phase II (Figure S3). Such a progressed monoclinic strain shifts the layers along their corrugation grooves and brings them closer (Figure S4), which amplifies intralayer distortions, clearly evidenced by a pronounced increase of σ^2^ and the departure of bond angles Pb–Br–Pb from their ideal value of 180° (Figure 2b and Table S9). Δ*d* (σ^2^) continues to decrease (increase) in phase II (Figure 2b and Tables S7 and S9). Note that although both angles systematically decrease, the change along [100] becomes much less pronounced compared to that along [210] (Figure 3). This anisotropy significantly increases the compressibility along *b*, resembling the behavior of the RT phase of DJ (API)­PbBr_4_.^38^ Our high-pressure Raman studies indicate that the lattice strain and the octahedra deformation anomalies coincide with the changes in hydrogen bonds of ACE^+^ cations (see below).

**Figure 3:**
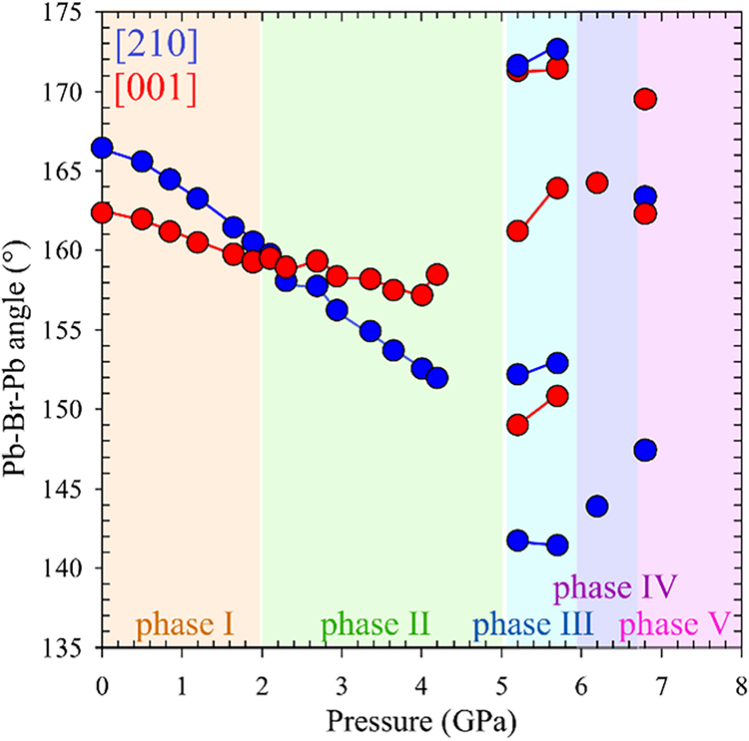
Evolution of the Pb–Br–Pb bond angles in ACE_2_PbBr_4_ as a function of pressure. The two plotted data sets correspond to bond angles measured along the [210] (blue circles) and [001] (red circles) crystallographic directions, illustrating the anisotropic nature of the compression.

Above 5 GPa, the continuous rise of the monoclinic β angle (Figure S3) is halted and additional reflections appear along c* in the reciprocal space (Figure S5), indicating the formation of another phase hereafter referred to as phase III (Figures 1 and S4). Phase III, again adopting the monoclinic space group *P*2_1_/*c*, has the unit cell tripled along the [*z*] axis, resulting in a unit-cell volume three times larger than those of phases I or II (Tables 1 and S6). Consequently, the number of formula units per unit cell (independent PbBr_6_ octahedra) increases from 4 to 12 (from 1 to 3), providing additional degrees of freedom for accommodating the compression strain. This improved efficiency of the strain accommodation mechanism in phase III is reflected by the fact that Δ*d* and σ^2^ significantly increase for two PbBr_6_ octahedra but decrease for the third octahedron, resulting in a slight increase of the mean values up to 6 GPa (Figures 2b and S6 and Tables S8 and S10). The origin of such a behavior is due to the widely open Pb–Br–Pb angles, connecting adjacent PbBr_6_ octahedra, driven by the relocation and reorientation of ACE^+^ cations into voids within the polyanion. Further compression pushes these cations deeper into the layer grooves.

Above 5.9 GPa, the additional reflections disappeared, indicating the PT to phase IV (Figures 1 and S4). Its *P*2_1_/*c* symmetry with the unit-cell parameters resembling those of phases I or II suggests a so-called reentry PT. However, the layers in phase IV accommodate the ACE^+^ cations tightly packed in the grooves within the corrugated layer. This position is additionally stabilized by strong charge-assisted HBs to the neighboring Br^–^ linkers, additionally deforming the inorganic layer. A significant increase in σ^2^ and an elongation of Pb–Br bonds are observed, manifested as a jumpwise increase in the Δ*d* parameter to the value nearly 3-fold higher than those in lower-pressure phases II and III, illustrating the interplay between cations and polyanions (Figures 2b and S6 and Tables S8 and S10). It is remarkable that in phase IV, the unit-cell parameters *a* and *c* reverse their lengths compared to phases I and II (Figures 2 and S2); the parameter *c* abruptly lengthens and the parameter *a* strongly shortens.

Phase V, observed at 6.8 GPa, adopts a triclinic space group *P*1̅ while maintaining the unit cell closely resembling those of phases I, II, and IV (Figure 1). The reduction of the symmetry constraints in ACE_2_PbBr_4_ phase V can thus be interpreted as a next step toward the improved accommodation of the compression strain. The penetration of ACE^+^ cations into the grooves within the polyanion requires an additional elongation of Pb–Br bonds and a wider opening of Pb–Br–Pb angles (Figure 3), which enhance the compressibility along the [001] direction. Additionally, a remarkable change in unit-cell dimensions is observed, as parameters *a* and *c* become very similar (Tables 1 and S6), despite the fact that the *a*-axis corresponds to the interlayer translation, while *c* runs along the grooves of the corrugated layers. Between phases IV and V, the length of the parameter *b* significantly increases, while parameters *a* and *c* weakly and significantly shrink below their lengths in phase IV (Figure 2a). It appears that further volume compression can be attributed mainly to the skewing of the unit-cell angles α and γ (Tables 1 and S6).

It can be noted that the isostructural PT between phases I and II, as well as the reentry PT to phase IV, marks the stability of the (110)-oriented RP-type frameworks. An analogous crystal of G_2_PbI_4_ (G^+^ denotes the guanidinium cation) was found to undergo an isostructural PT at a considerably lower pressure of 0.6 GPa;^51^ it appears that this change can be due to the larger grooves in the polyanion layers and the smaller size of G^+^ compared to ACE^+^ cations.

### Raman Study

The Raman spectra of ACE_2_PbBr_4_ are presented in Figure 4. The pressure dependence of the wavenumbers, described using a linear function ω­(*P*) = ω_0_ + α*P*, is shown in Figure 5, and values of wavenumber intercepts at zero pressure (ω_0_) and pressure coefficients (α = dω/d*P*), obtained from fitting of the experimental data by linear functions, are summarized in Table S11. Table S11 also lists the assignment of modes based on previous studies of acetamidinium and fomamidinium compounds as well as theoretical calculations for ACE^+^.^52–54^

**Figure 4:**
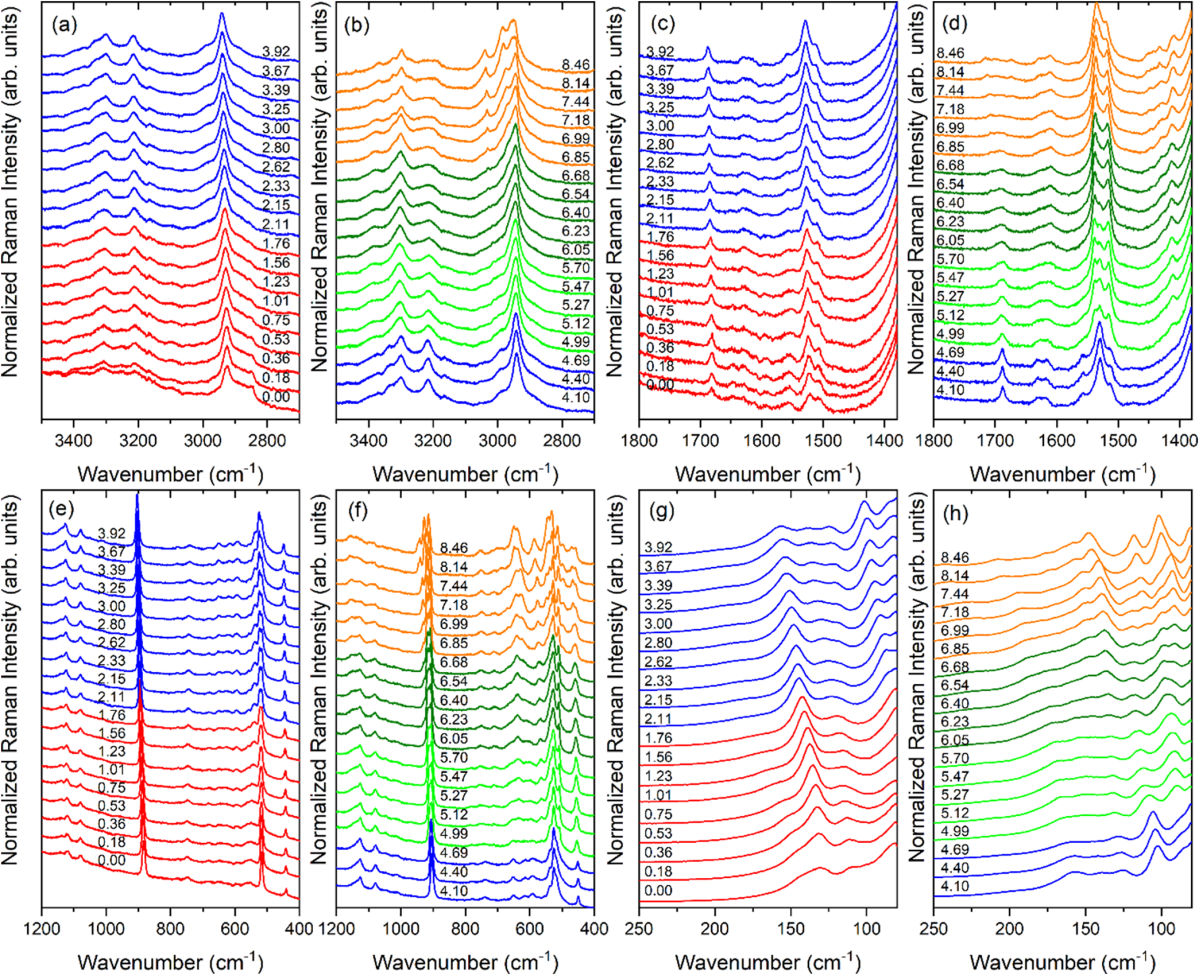
Raman spectra of ACE_2_PbBr_4_ under compression in the (a, b) 3450–2750, (c, d) 1800–1380, (e, f) 1200–400, and (g, h) 250–80 cm^–1^ ranges. Spectra in red, blue, green, olive, and orange correspond to phases I, II, III, IV, and V, respectively.

**Figure 5:**
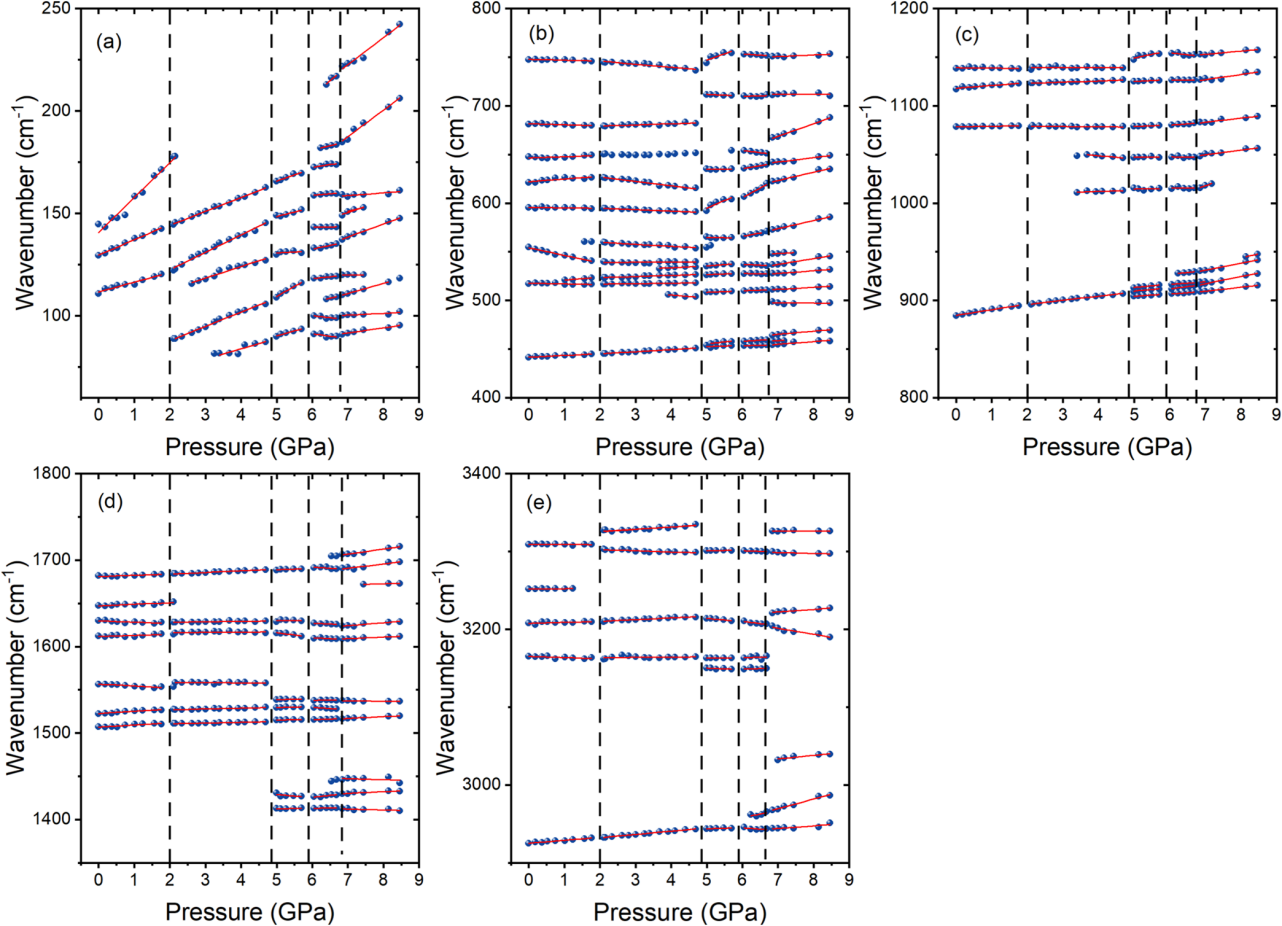
Wavenumber vs pressure plots of the Raman modes of ACE_2_PbBr_4_ for the compression experiment (circles) in the (a) 250–60, (b) 800–400, (c) 1200–800, (d) 1800–1350, and (e) 3400–2900 cm^–1^ ranges. The solid lines are linear fits on the data to ω­(*P*) = ω_0_ + α*P*. The vertical lines represent the pressure at which the PTs occur.

Raman spectra do not show any evidence of a pressure-induced PT up to 1.76 GPa (Figure 4). However, many modes of the NH_2_ groups exhibit a negative pressure dependence in the 0–1.76 GPa range, as seen in the ν­(NH_2_), δ­(NH_2_), ω­(NH_2_), and τ­(NH_2_) modes at ω_0_ = 3309.7 + 3165.2, 1629.8 + 1556.7, 747.7 + 681.6, and 595.5 + 554.2 cm^–1^, respectively, while for the majority of the other internal modes of ACE^+^, the pressure coefficients α are positive but small (Figure 5 and Table S11). This behavior indicates that pressure leads to pronounced strengthening of HBs and weak shortening of C–C and C–N bonds; i.e., compression strongly affects amine-framework interactions but has a weak effect on the internal structure of the ACE^+^ cation. Contrary to the internal modes, the lattice modes show a strongly positive pressure dependence with the coefficient α up to 17.1 cm^–1^GPa^–1^ (Table S11), indicating the strong compressibility of the inorganic framework.

Inspection of the spectra shows that above 2 GPa, the Pb–Br stretching band near 120 cm^–1^ (at 1.76 GPa) starts to split into two components. Many modes exhibit a significant change of the slopes (Figures 4g and 5) and in some cases, the negative (positive) pressure coefficients α in the 0–1.76 GPa range change to positive (negative) (Table S11). The observed changes are consistent with the isostructural character of the PT to phase II triggered by reorientation of the ACE^+^ cation and subsequent modification of the HB network, their strength and increased tilting of the PbBr_6_ octahedra.

On further compression, Raman spectra exhibit sudden changes when the pressure increases from 4.69 to 4.99 GPa, indicating the PT to phase III (Figure 4b,d,f,h). First, many internal modes split. For instance, the ν­(CC) mode at 907 cm^–1^ splits into the 904.4 + 909.1 + 913.1 cm^–1^ triplet, indicating that the number of unique ACE^+^ cations is three times larger compared to phases I or II. This observation is in agreement with tripling of the unit cell revealed by the X-ray diffraction data. Second, many internal modes exhibit sudden shifts. For instance, the δ­(CNC) band at 503.9 cm^–1^ shifts to 508.4 cm^–1^. This behavior is consistent with the first-order character of the PT and indicates that the PT leads to a significant increase of the amine-framework interactions, which affects the internal structure (bond lengths and angles) of ACE^+^. Interestingly, lattice modes do not show any significant shifts or splitting at the PT (Figures 4h and 5a), indicating that the PT does not lead to any significant distortion of the PbBr_6_ units. Third, bands related to vibrations of the NH_2_ groups exhibit a large increase of intensity and narrowing. As a result, some bands not visible at 4.69 GPa due to too weak intensities become apparent above 5 GPa (see, for instance, the τ­(NH_2_) bands near 565 and 635 cm^–1^ or the ρ­(NH_2_) bands near 1413 and 1432 cm^–1^ shown in Figure 4d,f). This behavior is consistent with a significant decrease of the motional freedom of ACE^+^ due to the presence of enhanced HBs. Overall, the observed changes indicate that this PT is triggered mainly by the reorientation and shifts of ACE^+^ cations, which in turn lead to the strengthening of HBs, distortion of ACE^+^ cations, and slowing down of their vibrational motions as well as tilting of the PbBr_6_ units.

When the pressure changes from 5.7 to 6.05 GPa, the lattice band at 94 cm^–1^ (the value at 5.7 GPa) starts to split and the intensity of the band ∼133 cm^–1^ starts to increase (Figure 4h). These changes are enhanced on further compression (Figure 4h), and analysis of the data reveals that several other lattice bands also split (Figure 5a). These changes indicate that ACE_2_PbBr_4_ experienced a PT to phase IV with distorted inorganic layers. In contrast to the lattice modes, Raman bands in the internal modes region do not show any clear evidence of the PT (Figure 4b,d,f), indicating that the internal structure of ACE^+^ is hardly affected by this PT. However, wavenumber vs pressure plots reveal that many internal modes exhibit a significant change of the slopes (Figure 5b–e and Table S11), indicating that the PT is associated with the reorientation of the ACE^+^ cations.

Above 6.7 GPa, a significant change of relative intensities and shifts of the lattice bands (Figures 4h and 5a) indicate that ACE_2_PbBr_4_ experienced a PT to a more distorted phase V. Very large splitting and appearance of new modes is also observed in the internal mode region (Figures 4b,d,f and 5b–e and Table S11). For instance, the number of the ν­(CN) and δ­(NCN) modes triples and doubles, respectively (Table S11). This behavior is consistent with X-ray diffraction data that revealed the lowering of the crystal symmetry to triclinic. In spite of such pronounced changes, a majority of Raman bands remain narrow up to 8.46 GPa, the highest pressure reached in our experiment. We do not observe, therefore, evidence of a static disorder, often reported for different 3D and some (001)-oriented lead halides.^29,33,55,56^

Pressure-induced structural changes are reversible, as evidenced by the reappearance of the bands during decompression and recovery of the ambient pressure phase at 0.08 GPa (Figure S7).

### Optical Properties

Figure 6a,b shows the PL and reflectance spectra measured at different temperatures. The PL spectra are dominated by a broad band at ∼2.0 eV. The very large full width at half-maximum (FWHM) of 1.14 eV and the very large Stokes shift of about 1.1 eV are distinct features of the STE emission, which is typical for lead halide perovskites with a large structural distortion, including 2D corrugated analogues.^14,15,23,24,57,58^ In addition to this emission, a weak emission at 3.35 eV is seen in the PL spectra at low temperatures. This emission is attributed to FE because at the same energy, an excitonic transition is observed in the reflectance spectrum (Figure 6b). The spectral position and broadening of the FE transition extracted from the reflectance spectra are shown in Figure 6d, and example fits and the formula used for fitting are given in Figure S8. As can be seen in Figure 6d, the PL band energies assigned to FE (open diamonds) spectrally match very well with the FE energy extracted from reflectance measurements (full diamonds).

**Figure 6:**
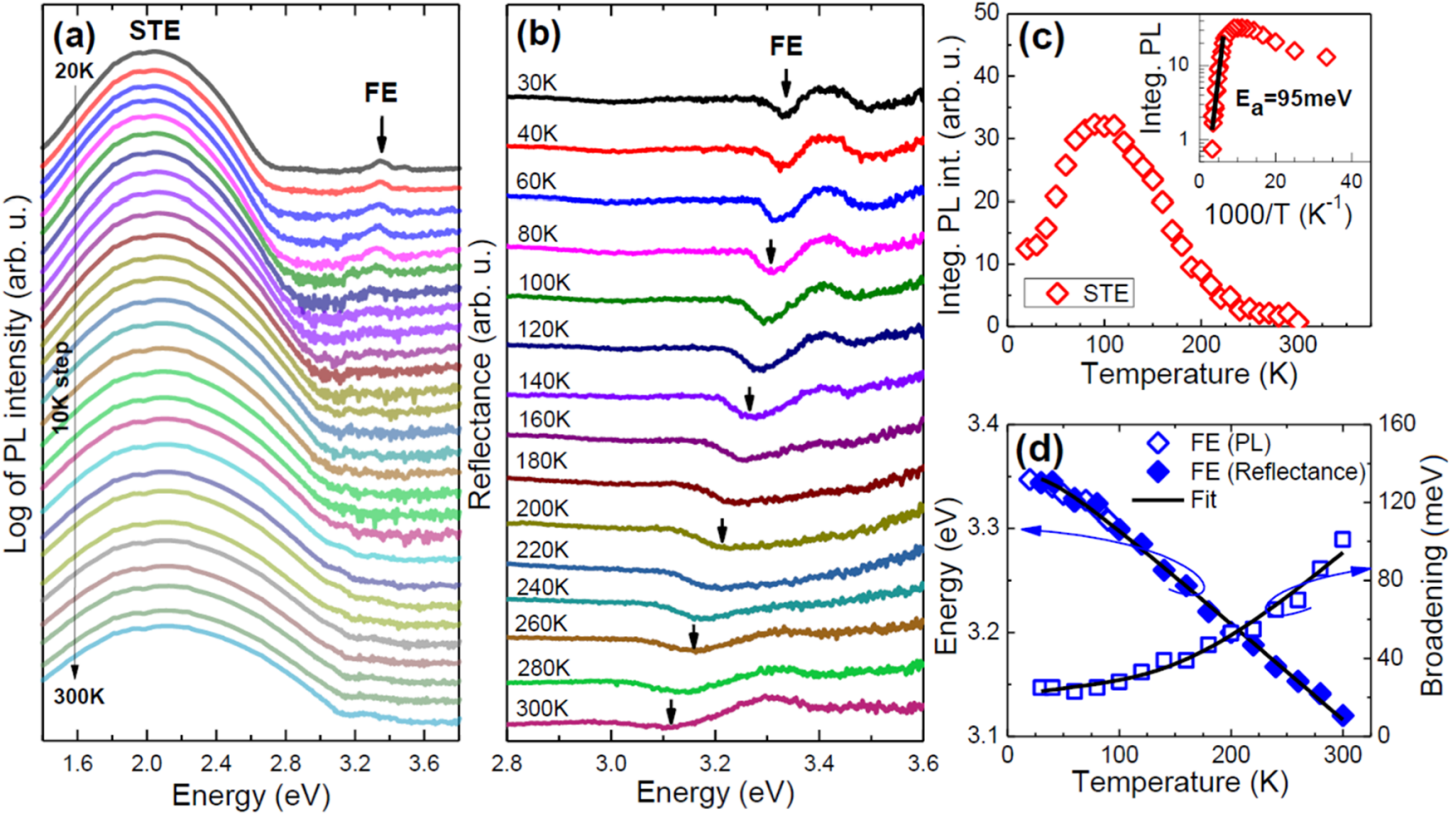
(a) PL spectra measured at different temperatures. (b) Reflectance spectra measured at different temperatures. (c) Integrated STE emission intensity and its analysis (inset). (d) Spectral position of the FE transition obtained from the PL (open diamonds) and reflectance (solid diamonds) spectra; broadening of the FE transition (open squares) observed in the reflectance spectra.

It is worth emphasizing here that the temperature evolution of the PL and reflectance spectra does not indicate the presence of a PT; i.e., this evolution is very systematic, and only continuous spectral shifts and an increase in broadening with increasing temperature can be seen. Therefore, the temperature dependence of the energy and FWHM of the FE transition can be analyzed over the full-temperature range with the same formula. The corresponding equations for this analysis are presented in the Supporting Information with appropriate comments on the determined parameters. The fitting curves are shown as solid black lines in Figure 6d.

The STE emission exhibits a noteworthy 43-fold intensity enhancement on cooling to 90 K, followed by a 2.6-fold intensity decrease on further cooling to 20 K (Figure 6c). From the temperature dependence of the STE intensity above 100 K, the activation energy (*E*_a_) was determined to be 95 ± 10 meV (the inset in Figure 6c), where the fitting formula used is as follows: $I\left(T\right)=I_{0}e^{E_{a}/\mathrm{kT}}$ , where *I*(*T*) is the temperature dependence of the integrated PL intensity, *I*_0_ is the intensity before the thermal quenching, and *kT* is the thermal energy. The obtained *E*_a_ is small compared to the spectral position of STE relative to the FE position (2.0 vs 3.35 eV) and is therefore attributed to the additional confinement of STE on deep donors (or acceptors) rather than to the exciton self-trapping energy, which in this case should be more than 1 eV. Knowledge of the emission intensity as a function of temperature allows us to estimate the internal quantum efficiency (IQE) as the ratio of the intensity at a given temperature (*I*_PL_(*T*)) to the intensity at a low temperature (*I*_PL_(20 K)) for which the quantum efficiency is close to 100% (IQE = *I*_PL_(*T*)/*I*_PL_(20 K) × 100%). This estimate is very often used for the active part of gallium nitride-based LEDs, i.e., InGaN/GaN quantum wells,^59,60^ and other III–V semiconductors. Using this approach, we determined the IQE at a few %, which suggests that we are dealing here with STEs additionally confined on deep donors (or acceptors), for which nonradiative recombination increases with increasing temperature. It is very typical for semiconductors that deep donors and acceptors are sources of nonradiative recombination. At low temperatures, their nonradiative nature is effectively suppressed, and excitons can additionally localize on them.^61^ However, as the temperature increases, the probability of exciton dissociation increases, and hence the contribution of nonradiative recombination increases, thus decreasing the IQE. The emission measurement results for ACE_2_PbBr_4_ and their analysis reveal a similar mechanism for STE recombination in this system; i.e., the self-trapping mechanism is responsible for the strong excitonic emission with a large Stokes shift, but the point defects, which are responsible for deep donors or acceptors, adversely affect the efficiency of this emission, which becomes evident at higher temperatures.

In the next step, we performed absorption-like (light transmission through the crystal) and PL measurements as a function of hydrostatic pressure at RT. Figure 7a shows the transmission spectra measured during compression (top panel) and decompression (bottom panel), and Figure 7b shows the selected spectra along with their analysis, based on which the absorption edge was estimated. Due to the scattered light passing outside the sample, which is typical for such measurements due to multiple reflections in the DAC (Figure 7b), calculating the absorption spectra from the transmission spectra leads to a significant underestimation of the absorption edge value. Moreover, for the correct determination of the absorption edge from the absorption spectra, the sample must be suitably thin (in this case, below 1 μm), which additionally complicates the measurements and is, therefore, rarely practiced in measurements under hydrostatic pressure. Therefore, in this case, to estimate the absorption edge, we extrapolated the linear decrease of the light transmission intensity through the crystal to zero, as shown by the arrows in Figure 7b. The energies read in this way are presented in Figure 7c together with the ranges of the individual crystallographic phases determined on the basis of structural studies (color backgrounds shown in Figure 7c). The obtained results clearly show that with the increase of hydrostatic pressure, the energy gap of ACE_2_PbBr_4_ narrows over the entire pressure range, but the pressure dependencies of this band gap narrowing in the individual phases are different. In the first approximation, it can be assumed that these are linear dependencies. With this assumption, the values of pressure coefficients were determined based on the linear fit (Figure 7c). It is worth adding that in spite of the significant shift of the band gap, no color change of the crystal was observed in the light transmitted to the naked eye, which is understandable due to changes in the absorption edge at the limit of human UV visibility.

**Figure 7:**
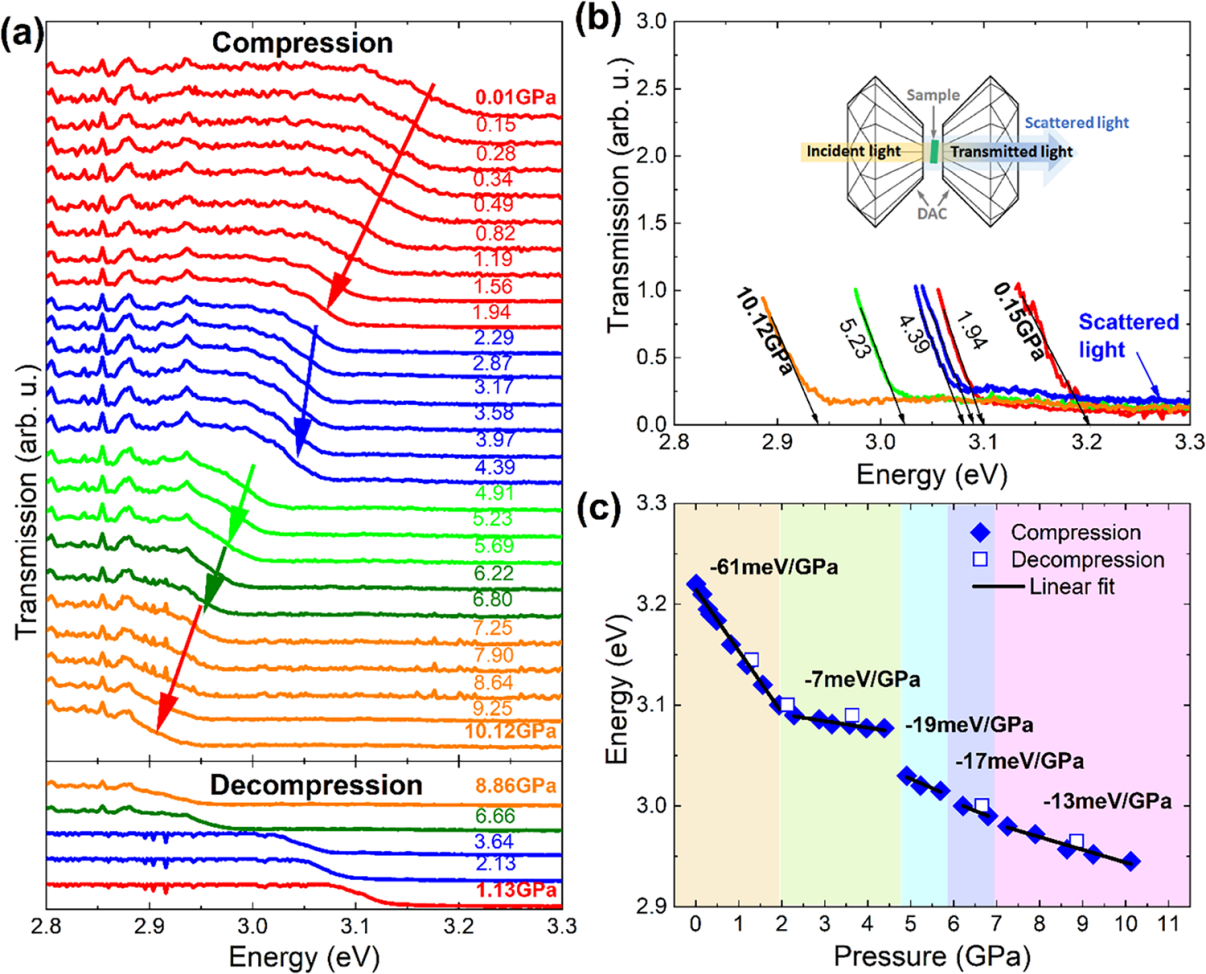
(a) Light transmission measurements through the ACE_2_PbBr_4_ crystal obtained upon compression to 10 GPa (top) and decompression (bottom panel). (b) Analysis of the selected transmission spectra with a sketch illustrating the scattered light resulting from multiple reflections in the DAC. (c) Absorption edge estimated from transmission measurements under hydrostatic pressure with linear fitting (black solid lines) and determined pressure coefficients for individual crystallographic phases (−61 ± 3, −7 ± 2, −19 ± 3, −17 ± 3, and −12 ± 2 meV/GPa).

Figure 7c shows that the PT near 2 GPa does not lead to any discontinuity in the band gap but leads to a large decrease of the pressure coefficient above 2 GPa. In lead halide perovskites, the increased overlap between Pb­(6s) and Br­(4p) atomic orbitals induced by the reduction in the Pb–X bond length results in a red shift of the band gap, whereas increased octahedral distortion (especially decreased Pb–X–Pb angles) has an opposite effect.^14,15,30,33,34^ The observed behavior near 2 GPa is, therefore, consistent with a lack of a significant deformation of PbBr_6_ octahedra and Pb–Br–Pb angles at this PT and the smaller compressibility of phase II along the *a* and *c* directions, as revealed by X-ray diffraction and Raman spectroscopy.

A sudden narrowing of the band gap is observed at the PT from phase II to phase III near 5 GPa. According to the X-ray diffraction and Raman data, this PT hardly affects Δ*d* and σ^2^ parameters but leads to a sudden decrease of the monoclinic β angle (Figure S3), triplication of the unit cell, strengthening of HBs, and a decrease of the average Pb–Br–Pb angles. The observed narrowing of the band gap can therefore be attributed to the shortening of Pb–Br bonds and the decrease of the average Pb–Br–Pb angles. The two remaining PTs near 5.9 and 6.7 GPa lead to weak discontinuities in the band gap, indicating that shortening of the Pb–Br bonds prevails over deformation of PbBr_6_ units.

Additionally, the absorption edge after decompression was plotted by open points shown in Figure 7c. Within the measurement uncertainties, the energies after decompression agree with those obtained during compression, which confirms that the observed changes in the transmission spectra are reversible.

Figure 8a shows the RT PL spectra measured under various hydrostatic pressures under compression up to 11.1 GPa (color lines) and under decompression (gray lines). The spectra show the presence of STE (band E3) and near-band-gap (NBE) emission composed of two narrow bands E1 and E2 that can be attributed to FEs and/or excitons containing large polarons in contrast to STEs composed of small polarons.^31^ The pressure-induced NBE evolution qualitatively agrees with the narrowing of the absorption edge estimated from transmission measurements as a function of the hydrostatic pressure. It can be seen that the PL spectra change continuously up to specific pressures at which PTs occur, and changes in the evolution of these spectra appear. Such PTs were identified around 2, 5, 5.9, and 6.7 GPa. As can be seen, the observed changes in the PL spectra are reversible upon pressure reduction with some hysteresis (Figure 8a), indicating the reversibility of the PTs.

**Figure 8:**
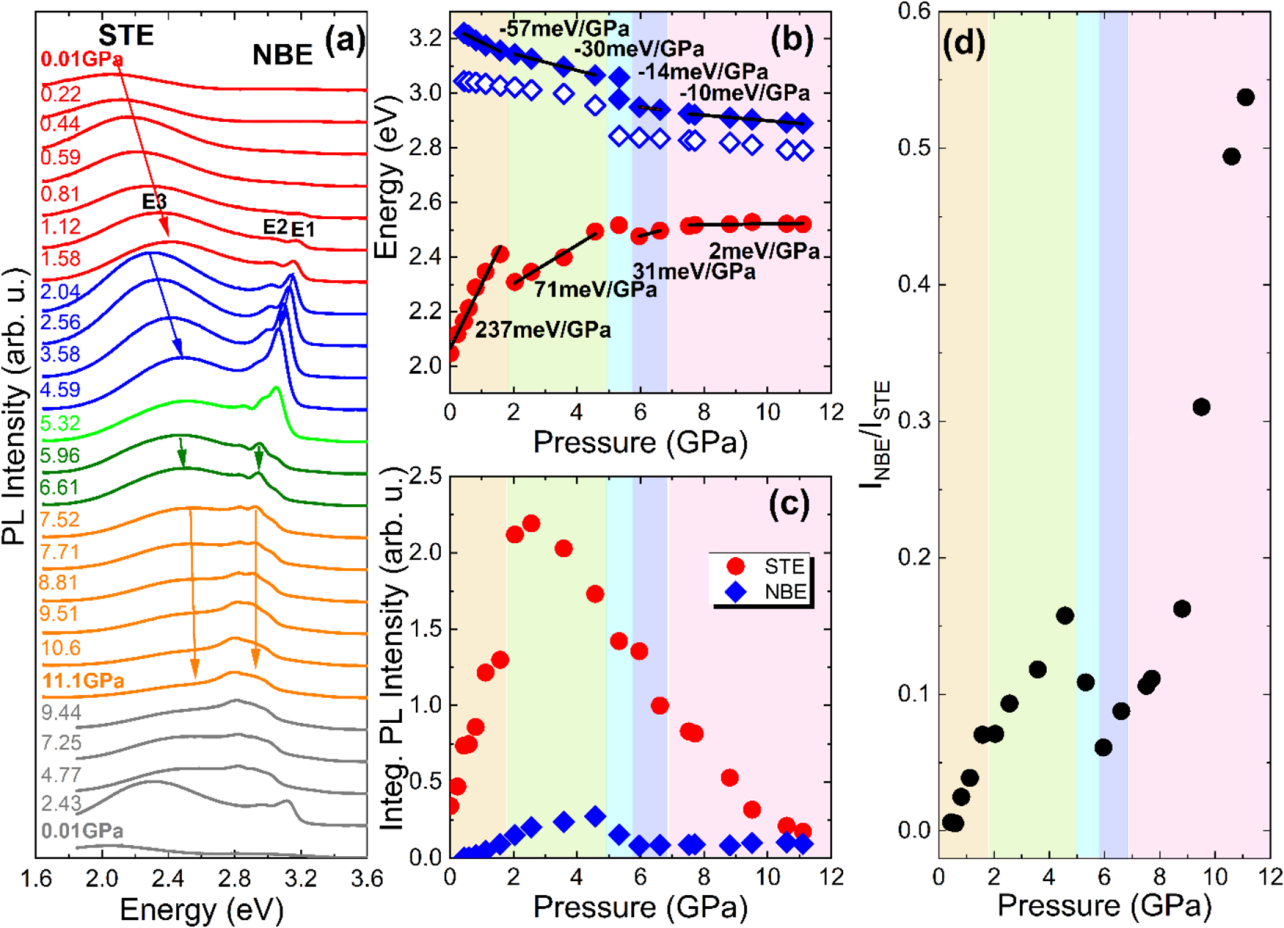
(a) RT PL spectra measured at various hydrostatic pressures under pressure upstroke (color lines) and downstroke (gray lines). (b) Spectral positions of STE (E3) and NBE (E1 and E2) bands as a function of hydrostatic pressure; the pressure coefficients are determined to be 237 ± 5, 71 ± 3, 31 ± 3, and 1 ± 0.2 meV/GPa for STE and −57 ± 3, −30 ± 3, −14 ± 2, and −10 ± 2 for E1 transition. (c) Integrated intensity of STE and NBE emissions as a function of hydrostatic pressure. (d) Ratio of NBE and STE intensity as a function of hydrostatic pressure.

To quantitatively analyze the evolution of the PL spectra with hydrostatic pressure, they were fitted with Gaussian peaks. Then, the spectral position of the bands and their intensities were analyzed, as seen in Figure 8b,c, respectively.

With the increase of pressure to 1.58 GPa, the STE emission shifts toward higher energies and its intensity increases (Figure 8a–c). Since X-ray diffraction study revealed a decrease of the Δ*d* parameter on compression, the observed intensity increase of the STE emission cannot be attributed to the increased octahedral distortion, as reported for many layered perovskites, but rather to the pressure-induced suppression of nonradiative recombination processes. Like STE emission, NBE is sensitive to hydrostatic pressure in the ACE_2_PbBr_4_ crystal, but this emission is not observed at 0.01 GPa and starts to be visible at 0.44 GPa. The E1 and E2 bands show a red shift and increase in intensity on compression to 1.58 GPa (Figure 8a–c). The observed linear dependence of the red shift confirms the assignment of the E1 and E2 bands to FEs. Note that the pressure dependence of the STE and NBE bands is opposite, resulting in a decrease of the Stokes shift on compression. Furthermore, an increase in the NBE intensity is more pronounced compared to the STE PL, as evidenced by the increase of the intensity ratio of these emission bands (Figure 8d). This behavior suggests that the increase in intensity of the E1 and E2 bands is at least partially related to pressure-induced detrapping from STEs to FEs. Since numerous literature data showed that in the layered lead halide perovskites, the Stokes shift and intensity of the STE band (NBE bands) increase (decrease) with an increasing degree of lattice distortion,^14,15,23,58^ the observed features of ACE_2_PbBr_4_ could be to some extent related to the above-mentioned decrease of the Δ*d* parameter on compression. However, very recently, the transfer from the STEs to FEs was also reported for (110)-oriented (API)­PbBr_4_, in spite of the increased Δ*d* on compression.^38^ It is therefore very likely that the observed detrapping can be at least partially attributed to the same mechanism as discussed in detail for (API)­PbBr_4_, i.e., to the reduced confinement in the direction of the corrugated structure (across the folds of the corrugated polyanion) on compression, resulting in the increased electronic dimension and the appearance of a barrier for STE formation, allowing more carriers to retain as FEs.^38^ For further research on the exciton trapping/detrapping phenomenon, time-resolved PL measurements as a function of temperature would be advisable, but such measurements are beyond the scope of this article.

The PT to phase II is evidenced when the pressure increases to 2.04 GPa by the discontinuous shift of the STE band to a lower energy, the abrupt increase of its intensity, and the Stokes shift. The lack of any discontinuities in the shifts of the NBE band energies at the PT pressure indicates that the behavior of the STE emission can be related to weak changes in the tilts of the PbBr_6_ octahedra without their significant deformation. Note that the intensity of the NBE bands does not show any decrease at the PT pressure (Figure 8c), contrary to (API)­PbBr_4_, for which a very pronounced quenching of the FE emission was observed just above the isostructural PT pressure reported at 2.7 GPa.^38^ This behavior points to significant differences in the isostructural PT mechanism of both compounds and properties of the high-pressure phases. Indeed, in the case of (API)­PbBr_4_, the bond-length distortion Δ*d* significantly increased and compressibility along the corrugation direction decreased above 2.7 GPa,^37^ while in ACE_2_PbBr_4_, an opposite behavior is observed. With a further increase of pressure to 4.59 GPa, a clear decrease in the STE emission intensity is visible but the STE band continues to shift toward higher energies, although with a smaller slope (71 vs 237 meV/GPa, Figure 8b). A decrease in the slope is also observed for the FE emission (−30 vs −57 meV/GPa, Figure 8b). However, contrary to the STE PL, the NBE intensity increases on further compression in the 2.05 to 4.59 GPa range (Figure 8c). The observed features indicate that compression of phase II leads to a further decrease of the electron–phonon coupling strength due to the further decrease of the Δ*d* parameter and reduction of the spacing between lead atoms, especially along the corrugated direction, as revealed by the X-ray diffraction data, thereby reducing confinement in this direction.

On further compression, some changes in the PL spectra become evident at 5.32 GPa. First, the intensity of the NBE decreases (Figure 8c). Second, a new red-shifted band appears at 2.844 eV (Figure 8a,b). The observed changes could be attributed to the onset of the PT from phase II to phase III. However, the presence of three NBE bands at 5.32 GPa and the fact that the band gap suddenly narrowed at this PT (see Figure 7c) indicate the coexistence of two phases (II and III) at this pressure point.

The next PT to phase IV is expected to be near 5.9 GPa. Optical studies show that the STE (E1) emission blue shift (red shift) on compression of phase IV is no longer so strong (the pressure coefficient is 31 meV/GPa (−14 meV/GPa)) and that the NBE bands do not show any clear discontinuities at the PT pressure (Figure 8a,b). However, a weak discontinuous shift to a lower energy is observed for the STE emission. Furthermore, the I_NBE_/I_STE_ ratio decreases (Figure 8d). This behavior indicates that the PT is associated with a rather weak increase of the octahedral distortion. On further compression of phase IV from 5.96 to 6.61 GPa, the Stokes shift decreases and the *I*_NBE_/*I*_STE_ ratio increases, indicating a further decrease of the electron–phonon coupling strength in phase IV on compression.

The last PT to the triclinic phase expected near 6.7 GPa does not lead to any significant changes in the PL properties. Analysis of the data shows a further decrease in the pressure coefficients, a decrease of the STE emission intensity, and an increase of the I_NBE_/I_STE_ ratio above 6.7 GPa (Figure 8). Thus, the behavior of phase V resembles that of phase IV.

To explain the FE and STE emissions as well as the interrelationship between these emissions, a configuration diagram is often used.^31,62–65^ A corresponding diagram was also proposed in our case (Figure 9) to explain the red shift and blue shift for NBE and STE, respectively, as well as the increase in the ratio of the NBE emission intensity to STE emission with increasing pressure. In our diagram, the NBE is represented by FE and the discussed changes are within the same crystallographic phase. The key element in this diagram is the pressure-induced shift of the STE energy minimum on both the energy scale (this leads to the emission red shift) and the configuration coordinate scale (Q-scale), which explains the observed STE blue shift. Another important element in this diagram is the FE to STE transfer, which is symbolically marked by a yellow arrow and whose efficiency decreases with increasing pressure due to the change in the potential barrier and the position of the energy minima for FE and STE. Our studies have shown that this transfer is very sensitive to hydrostatic pressure. We expect that the temperature also plays a significant role in this transfer, so measurements of the activation energy for STE and NBE at selected pressures could be of great interest in further studies.

**Figure 9:**
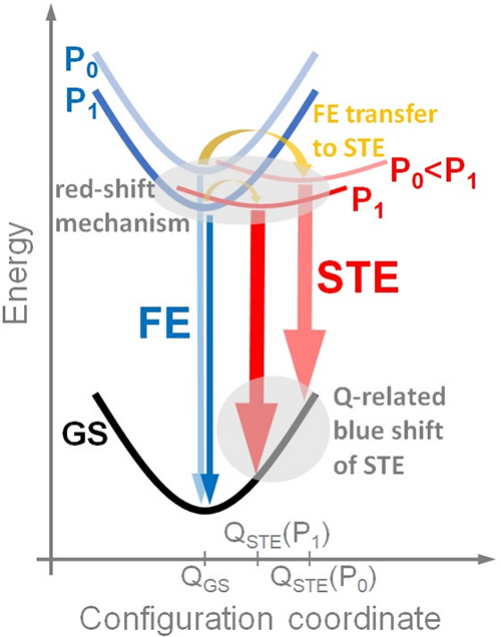
Configuration diagram explaining the red shift and blue shift of the FE and STE emission in ACE_2_PbBr_4_ and the changes in the mutual intensity between FE and STE. GS is the ground state for this system. *P*_0_ and *P*_1_ correspond to pressures where *P*_1_ > *P*_0_.

Summarizing the proposed phase diagram, quantitative changes can be expected after a PT (i.e., a change in the shift factor for the FE and STE dispersions in the presented diagram), but phase changes can also be introduced, i.e., a discontinuous shift for STE, which may be indicated by a discontinuity in the STE emission peak position at ∼2 GPa. In addition, it is worth mentioning that exciton binding energies (FE and STE) can change with increasing pressure, which also affects the FE and STE spectral shift. However, we do not expect that in the studied pressure range, changes in the exciton binding energy will be crucial for the observed spectral shifts. To date, there are few reports on this topic for semiconductors. For WS_2_, it was recently reported that the contribution to the spectral shift of excitonic emission with pressure resulting from changes in the exciton binding energy is rather small.^66^

The emission color showed a strong piezochromism, spanning from orange-yellow to greenish-blue (Figure 10). However, it should be noted that we are dealing here with the presence of a fairly broad emission band, and therefore, we are not referring to pure RGB colors but only to the dominant color resulting from the spectral position of the PL bands.

**Figure 10:**
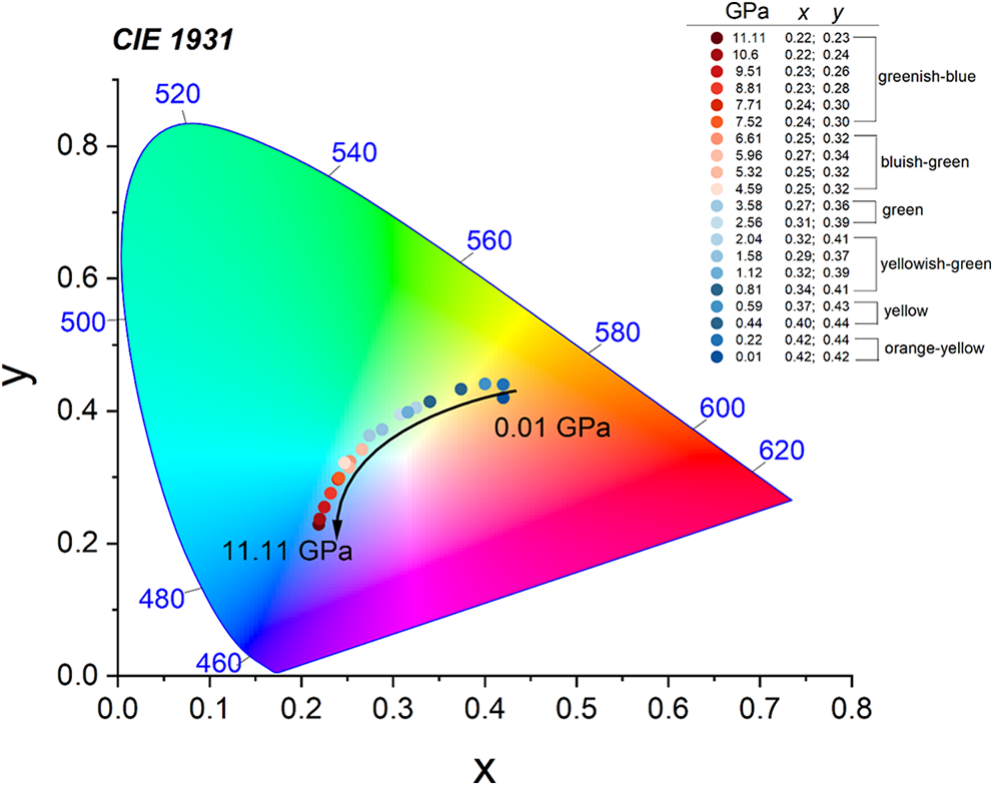
CIE coordinates of ACE_2_PbBr_4_.

So far, hydrostatic pressure emission tuning has been demonstrated for commercial devices, such as laser diodes based on III–V materials,^67,68^ which are characterized by very narrow wavelengths and which enable the production of pure colors. However, pure RGB colors are not always required in light emitters, and in this context, the tested material may be of interest. In general, perovskite LEDs are not yet as advanced as those for III–V materials, but the results of hydrostatic pressure emission tuning for ACE_2_PbBr_4_ indicate that this material has potential in this area, as well.

## Conclusions

In summary, we engineered a 2D nanorange layer system of the RP (110)-oriented ACE_2_PbBr_4_ perovskite and investigated the effect of external pressure on its structural, phonon, and optical properties in a very broad pressure range (0.01–11.11 GPa). We observed several spectacular changes in the PL of this compound under compression such as the simultaneous increase in intensity of both STE and FE emissions in the low-pressure regime, the sudden increase of the STE intensity near 2 GPa followed by its continuous decreasing above 2 GPa, and the sudden decrease of the FE intensity near 5–6 GPa as well as the red shift of the FE band near 5 GPa. We elucidated the physicochemical mechanism of the pressure-induced changes in this compound through X-ray diffraction and Raman studies. In particular, the very rare phenomenon of STE to FE transfer was attributed to the decreased bond-length distortion, contrasting with the increased octahedral distortion reported in most previous studies on lead halide perovskites, and the reduction of the spacing between the adjacent lead atoms, especially along the zigzag direction, thereby reducing electron confinement. The observed optical anomalies near 2, 5, and 5.9 GPa were attributed to pressure-induced isostructural PTs between *P*2_1_/*c* phases. Based on X-ray diffraction and Raman data, we were able to show that the PT near 2 GPa is associated with the reorientation of ACE^+^ cations accompanied by the weak tilting and distortion of the PbBr_6_ octahedra, which leads to a 3-fold increase of the unit cell volume near 5 GPa and strongly affects the internal structure of ACE^+^ cations due to the increase of the HB strength, whereas the PT near 5.9 GPa leads to the distortion of the inorganic layers. X-ray diffraction and Raman data also revealed the fourth PT near 6.7 GPa associated with further distortion of the inorganic framework and a symmetry decrease to triclinic, which has not led to any apparent changes in the optical properties.

X-ray diffraction also showed that ACE_2_PbBr_4_ exhibits nearly isotropic compressibility in the low-pressure regime, followed by a pronounced increase in the zigzag direction above 2 GPa, exceeding compressibility in the layer-stacking direction. The energy gap of this crystal narrows with increasing hydrostatic pressure. Overall, this work shows that ACE_2_PbBr_4_ is a rare example of a layered lead halide exhibiting a pressure-induced reduction of lead bromide octahedral distortion, transfer from STEs to FEs, and remarkable tunability of the emission color. It also shows that the mechanical behavior of this (110)-oriented RP perovskite on compression contrasts sharply with the behavior of the (001)-oriented perovskites. Although the behavior of this RP perovskite bears similarities to the behavior of (110)-oriented DJ (API)­PbBr_4_, mechanisms of the isostructural PTs occurring in these compounds are not the same. ACE_2_PbBr_4_ exhibits a more pronounced pressure-induced polymorphism (4 PTs vs 1), and the pressure dependence of the STE and FE band positions and intensities are different. This work provides a strategy to achieve efficient and strongly tunable emission by pressure-induced structural changes in layered perovskites and highlights the importance of organic cations in the regulation of the mechanical behavior, polymorphism, and PL properties of (110)-oriented perovskites. It appears that the nanospaced interlayer regions of (110)-oriented RP ACE_2_PbBr_4_ are ideally suited for the chemical engineering of strongly tunable and highly anisotropic materials desired for the deposition of films with optoelectronic, thermal, and mechanical properties.

## Supplementary Material





## Notes

Additional experimental details including crystallographic data, Raman wavenumbers and assignment, and analysis of reflectance spectra. (PDF)
Additional data. (CIF)
