# Supplementary material for: Pressure-Induced Detrapping from Self-Trapped Excitons to Free Excitons toward Enhanced Emission and Piezochromism in Ruddlesden–Popper (110)-Oriented Perovskites
Source: ACS Appl Mater Interfaces. 2025 Oct 9;17(42):58452–66. doi: 10.1021/acsami.5c14096 (PMC12557188; doi:10.1021/acsami.5c14096)
Supplement: Supplementary file 1 [file am5c14096_si_001.pdf]

Supporting Information for

Pressure-Induced Detrapping from Self-Trapped Excitons to Free Excitons Toward Enhanced Emission and Piezochromism in Ruddlesden-Popper (110)-Oriented Perovskite

Mirosław Mączka,*[a] Szymon Sobczak,[b] Kinga Roszak,[b] Daniel Linhares Militão Vasconcelos,[c] Filip Dybała,[d] Artur P. Herman,[d] Robert Kudrawiec,[d] Andrzej Katrusiak*[b] and Paulo T. C. Freire[e]

[a]*Institute of Low Temperature and Structural Research, Polish Academy of Sciences, Okólna 2, 50-422 Wroclaw, Poland*

[b] *Department of Materials Chemistry, Faculty of Chemistry, Adam Mickiewicz University, Uniwersytetu Poznańskiego 8, 61–614, Poznań, Poland*

[c]*Faculdade de Educação, Ciências e Letras do Sertão Central, Universidade Estadual do Ceará, Quixadá-CE, Brazil*

[d]*Department of Semiconductor Materials Engineering, Faculty of Fundamental Problems of Technology, Wrocław University of Science and Technology, Wybrzeże Wyspiańskiego 27, 50-370 Wrocław, Poland*

[e]*Departamento de Fisica, Universidade Federal do Ceara, P.O. Box 6030, 60455-970, Fortaleza, Brazil*

e-mail: m.maczka@intibs.pl, katran@amu.edu.pl

Contents

1. **Ambient pressure Raman** 2
2. **Compressibility of ACE_2PbBr_4** 3
3. **X-Ray diffraction data** 8
a. **Calculation of the distortion parameters Δ and σ** 11
4. **Raman Studies** 16
5. **Optical Studies** 20
6. **References** 22

1. Ambient pressure Raman

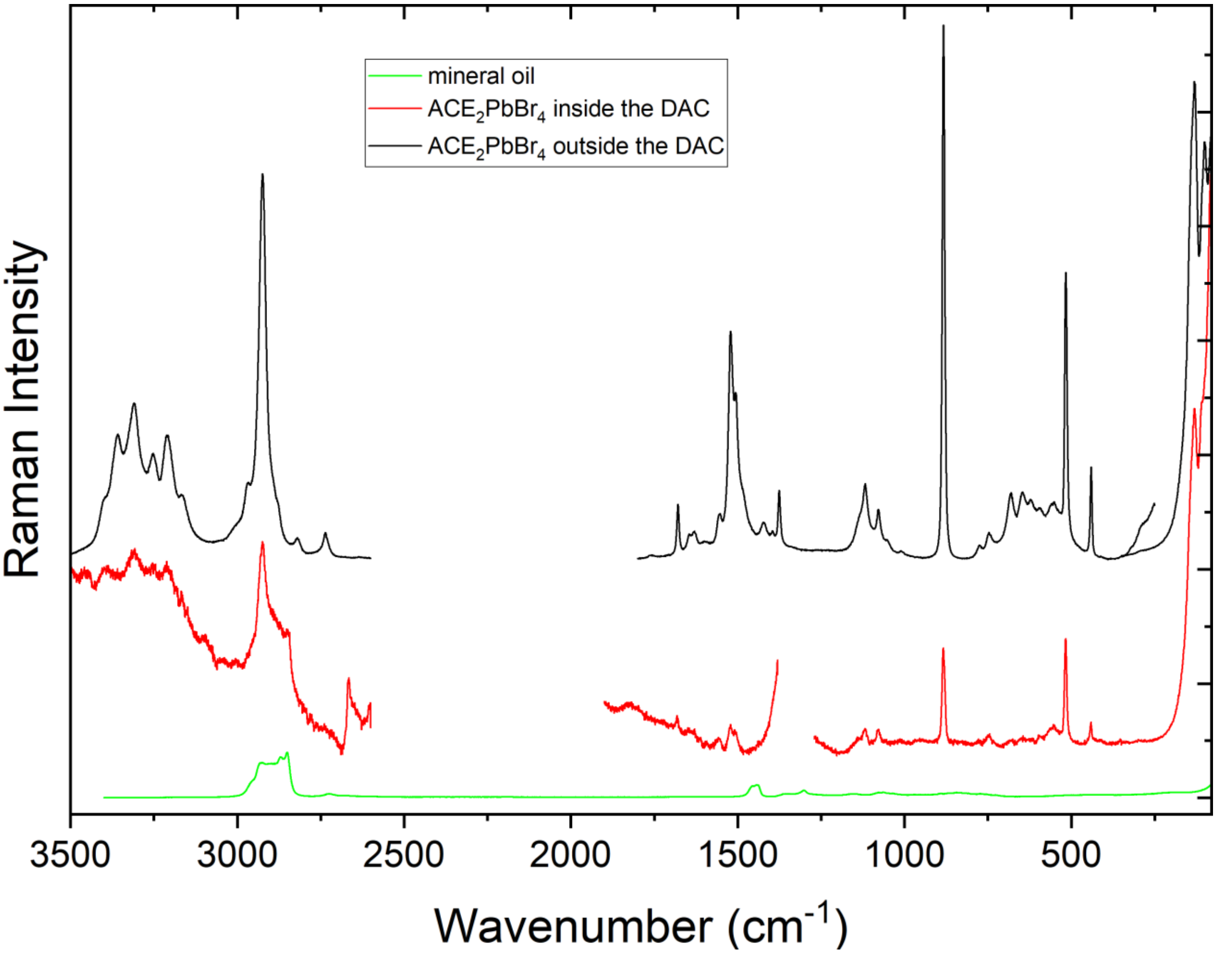

Figure S1. Raman spectra of mineral oil and ACE_2PbBr_4 outside DAC, and ACE_2PbBr_4 inside DAC at ambient pressure.

2. Compressibility of ACE$_2$PbBr$_4$

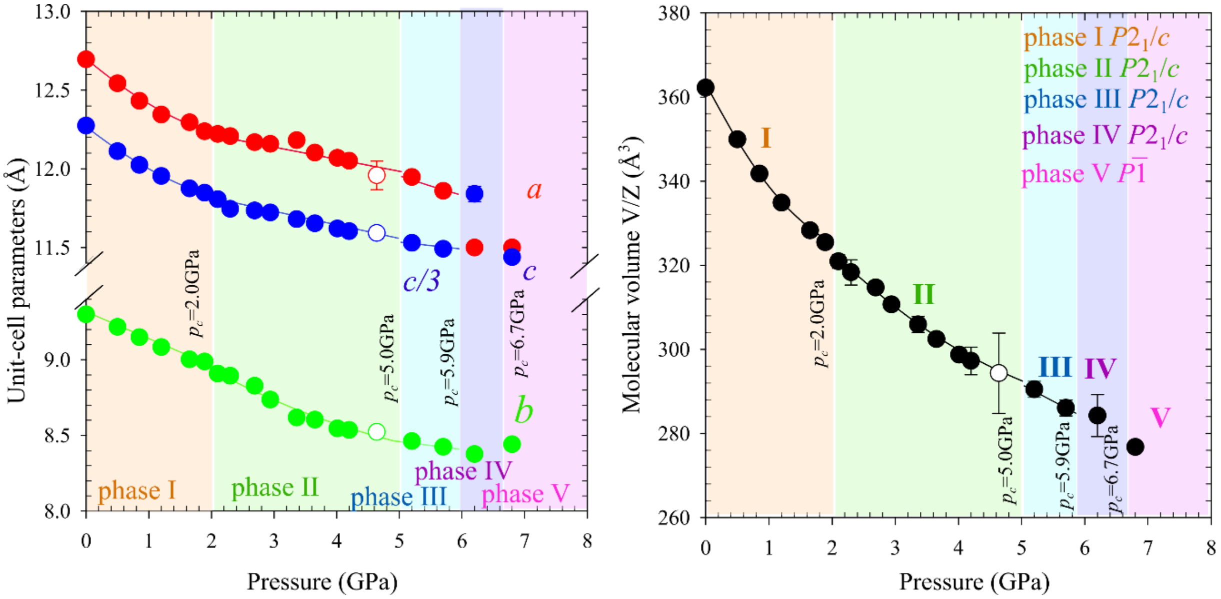

Figure S2. Pressure dependence of the unit-cell parameters (left) and normalized molecular volume V/Z (right) for the studied compound. Phases I–V are indicated with colored backgrounds corresponding to their respective stability ranges at RT. The critical transition pressures, p_c, are marked with vertical dashed lines. The open symbol at 4.7 GPa represents the measurement performed in Daphne 7575 oil, near its hydrostatic limit (cf. Experimental methods). Measurements above 5 GPa were conducted using a 1:1 mixture of *n*-pentanol and *iso*-pentanol as the pressure-transmitting medium.

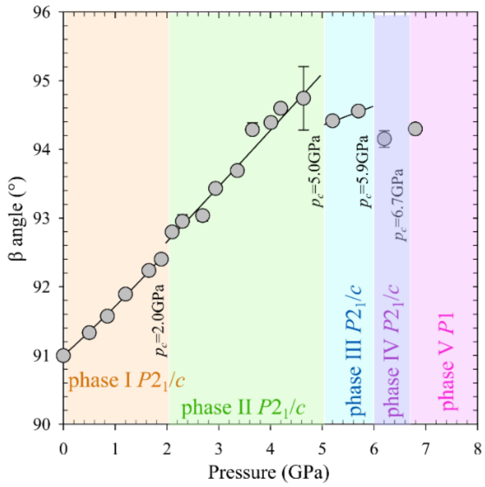

Figure S3. Pressure-induced changes in the monoclinic beta angle. The ESD, if not visible, are smaller than points.

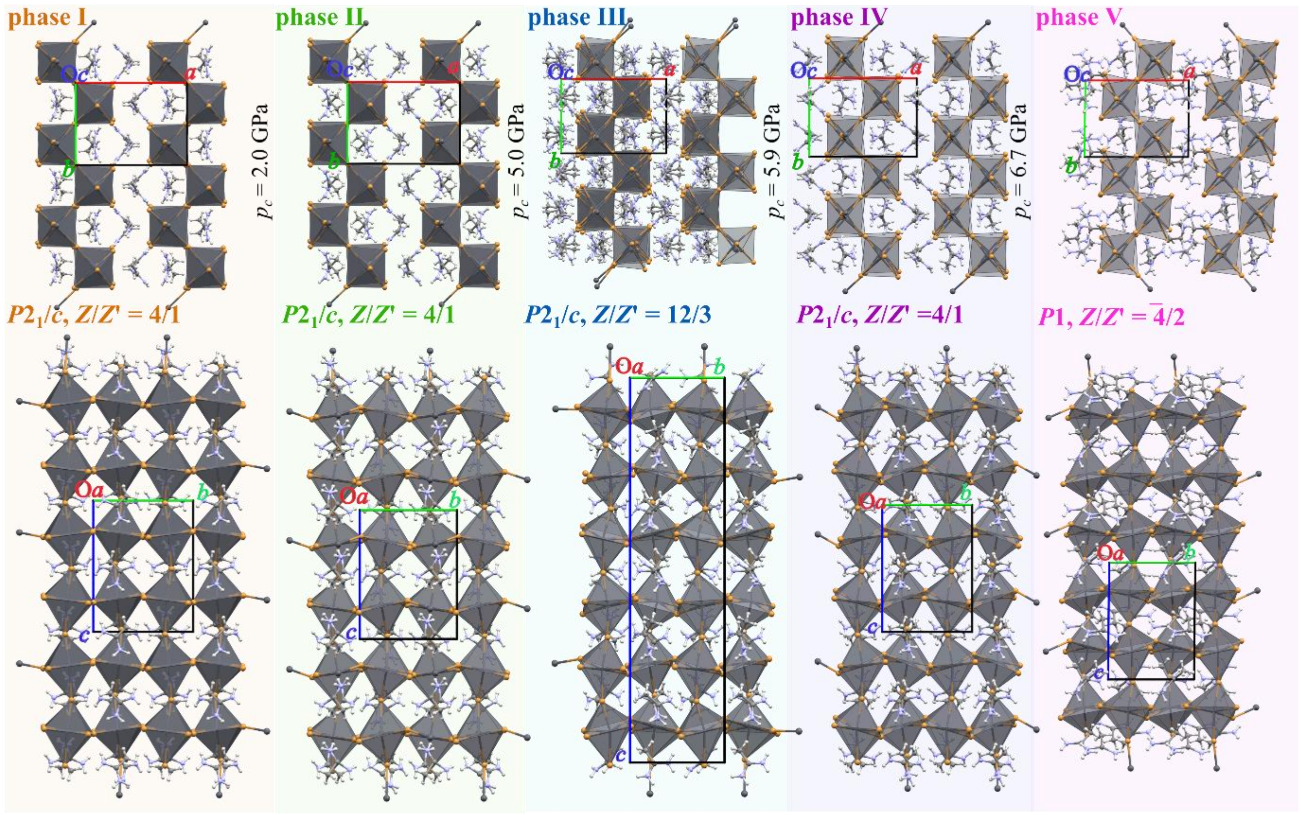

Figure S4. Crystal structures of the ACE_2PbBr_4 phases I - V at different orientations (top) viewed along the *c* direction, and (down) along the *b* direction. Atoms are color-coded: gray (carbon), white (hydrogen), blue (nitrogen) within the ACE^+ cation; orange (bromide linkers), and black (Pb^{2+} centers) for the atoms constituting the polyanionic layers.

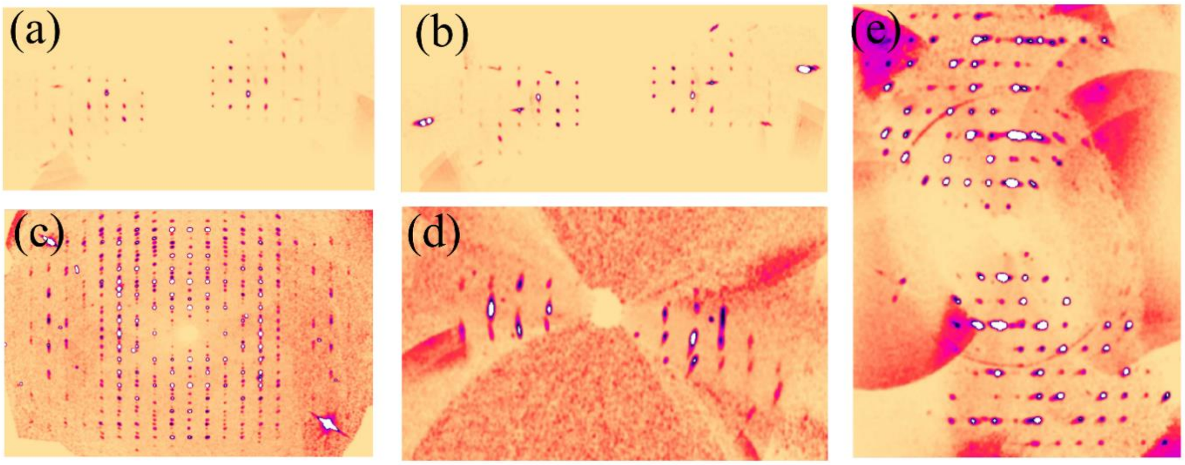

Figure S5. The reconstructed hk0 layer from the measurements for (a) phase I at 0.5 GPa; (b) phase II at 2.1 GPa; (c) phase III at 5.2 GPa; (d) phase IV at 6.2 GPa and (e) phase V at 6.8 GPa showing the increased number of reflections leading to elongation of the unit-cell direction *c* in phase III.

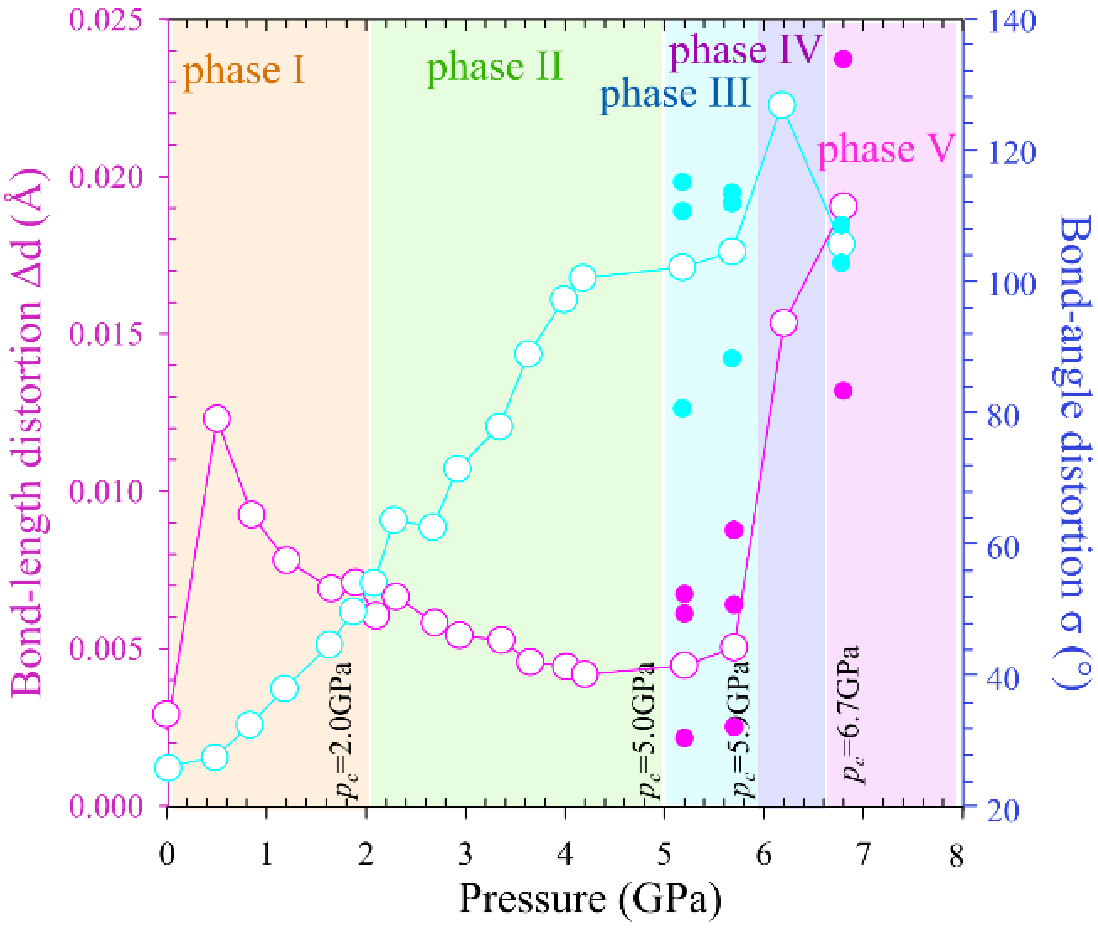

Figure S6. The $PbBr_6$ octahedra distortion measured by mean (open symbols) and individual (closed circles for phase III and V) bond-length deviation Δd (plotted in pink) and bond-angle variance σ^2 (blue).

Table S1. Compressibility related to crystallographic axes calculated for ACE_2PbBr_4 phase I calculated in the range between 0.5 GPa and 1.89 GPa with Birch-Murnaghan coefficients calculated with program PASCal.[1,2]

			Direction			Empirical parameters			
Axes	K (TPa^{-1})	σK(TPa^{-1})	a	b	c	ε_0	λ	P_c	ν
X_1	23.5547	2.4063	0.7882	0.0	0.6154	31.9804	-31.9802	-0.4985	0.0012
X_2	18.9003	31.7756	0.0	1.0	0.0	27.3884	-27.384	-0.6251	0.0012
X_3	8.95	0.6037	-0.5833	0.0	0.8123	16.1826	-16.1917	0.1355	0.0006
V	51.3727	4.6386							

Birch-Murnaghan Coefficients

	B_0 (GPa)	σB_0 (GPa)	V_0 (Å^3)	σV_0 (Å^3)	B'	σB'	P_c (GPa)
2nd	14.0276	1.5508	1443.3886	10.9165	4.0	0.5701	0.0

Compressibility coefficients

P (GPa)	K_1 (TPa^{-1})	K_2 (TPa^{-1})	K_3 (TPa^{-1})	σK_1 (TPa^{-1})	σK_2 (TPa^{-1})	σK_3 (TPa^{-1})
0.5	38.864	29.8027	24.8953	13.9418	181.2645	7.7695
0.85	28.7877	22.739	12.7053	2.6583	22.1118	1.007
1.20	23.5547	18.9003	8.95	2.4063	31.7756	0.6037
1.65	19.2418	15.6439	6.5593	2.5475	7.2819	0.6547
1.89	16.2644	13.3452	5.1768	4.2249	51.558	1.0397

Table S2. Compressibility related to crystallographic axes calculated for ACE_2PbBr_4 phase II calculated in the range between 2.10 GPa and 4.2 GPa with Birch-Murnaghan coefficients calculated with program PASCal.

			Direction			Empirical parameters			
Axes	K (TPa^{-1})	σK(TPa^{-1})	a	b	c	ε_0	λ	P_c	ν
X_1	19.0582	0.2504	0.8252	0.0	0.5649	66.4239	-66.4397	1.7775	0.0004
X_2	17.6395	5.4794	0.0	1.0	0.0	0.0662	-0.001	-83.6792	19.4741
X_3	0.0436	4.073	-0.5795	0.0	0.815	-0.0015	-0.0002	2.3187	12.0122
V	39.4198	2.3068							

Birch-Murnaghan Coefficients

	B_0 (GPa)	σB_0 (GPa)	V_0 (Å^3)	σV_0 (Å^3)	B'	σB'	P_c (GPa)
2nd	12.3723	0.8028	1476.4851	13.1092	4.0	0.7784	0.0

Compressibility coefficients

P (GPa)	K_1 (TPa^{-1})	K_2 (TPa^{-1})	K_3 (TPa^{-1})	σK_1 (TPa^{-1})	σK_2 (TPa^{-1})	σK_3 (TPa^{-1})
2.10	51.435	15.2629	0.0	63.757	0.2005	0.0
2.69	30.5848	16.5227	0.0	8.3324	0.2171	0.0008
2.94	24.0096	17.429	0.0001	8.3611	0.229	0.0795
3.36	17.6395	19.0582	0.0436	5.4794	0.2504	4.073
3.65	14.9086	20.266	0.6521	4.7079	0.2662	9.9668
4.01	12.5054	21.8663	9.0974	5.6682	0.2873	32.7522
4.2	11.525	22.7583	29.3818	6.4564	0.299	119.7127

Table S3. Summary of pressure-dependent axial and volumetric compressibilities for phases I–III. Compressibilities along the principal crystallographic directions were calculated using the general relation $\beta_x = -(1/x)(\partial x/\partial p)$, where x represents the unit-cell parameters (a, b, c) or volume V. Polynomial functions were fitted to the pressure evolution of the unit-cell dimensions in phases I and II using a least-squares approach, while for phase III, a linear fit was applied. The derived compressibility values are listed alongside the fitted equations.

Phase	function fitted along [100]	β_a	function fitted along [010]	β_b	function fitted along [001]	β_c	function fitted to volume change	β_V
I	$\beta a_I(p)$=12.70(1) – 0.32(6)p – 0.00(9)p^2+0.02(3)p^3	βa_I(1.89GPa) = 0.01979 GPa^{-1}	$\beta b_I(p)$=9.30(1) – 0.08(5)p – 0.19(7)p^2+0.07(2)p^3	βb_I(1.89GPa) = 0.0184 GPa^{-1}	$\beta c_I(p)$=12.272(8) + 0.32(4)p – 0.01(6)p^2+0.02(2)p^3	βc_I(1.89GPa) = 0.0191 GPa^{-1}	$\beta V_I(p)$=362.2(3) – 22(1)p – 6.1(19)p^2+3.9(7)p^3	βV_I(1.89GPa) = 0.0598 GPa^{-1}
II	$\beta a_{II}(p)$=11(3) + 0(2)p – 0.1(7)p^2+0.00(7)p^3	βa_{II}(4.20GPa) = 0.0129 GPa^{-1}	$\beta b_{II}(p)$=9(1) – 0.3(18)p – 0.03(36)p^2+0.01(3)p^3	βb_{II}(4.20GPa) = 0.0185 GPa^{-1}	$\beta c_{II}(p)$=12.5(9) – 0.5(8)p + 0.1(3)p^2 – 0.006(25)p^3	βc_{II}(4.20GPa) = 0.0076 GPa^{-1}	$\beta V_{II}(p)$=356(12) – 15(11)p – 1(3)p^2+0.3(3)p^3	βV_{II}(4.20GPa) = 0.0377 GPa^{-1}
III	$\beta a_{III}(p)$=12.8622 – 0.1760p	βa_{III}(5.70 GPa)= 0.01480 GPa^{-1}	$\beta b_{III}(p)$=8.8563 – 0.0758p	βb_{III}(5.70 GPa) = 0.090 GPa^{-1}	$\beta c_{III}(p)$=11.9228 – 0.0758p	βc_{III}(5.70) = 0.0198 GPa^{-1}	$\beta V_{III}(p)$=336.6112 – 8.8660p	βV_{III}(5.70GPa) = 0.0310 GPa^{-1}

3. X-Ray diffraction data

Table S4. Detailed crystallographic data for ACE_2PbBr_4 at high-pressure (0.5-2.1 GPa) and RT.

Pressure	**0.50 GPa**	**0.85 GPa**	**1.20 GPa**	**1.65 GPa**	**1.89 GPa**	**2.10 GPa**
	phase I	phase I	phase I	phase I	phase I	phase II
CCDC numbers	2369881	2369882	2369883	2369884	2369885	2369886
Crystal system	monoclinic	monoclinic	monoclinic	monoclinic	monoclinic	monoclinic
Space group	$P2_1/c$	$P2_1/c$	$P2_1/c$	$P2_1/c$	$P2_1/c$	$P2_1/c$
Unit cell dimensions						
a (Å)	12.542(9)	12.431(9)	12.344(9)	12.294(10)	12.237(12)	12.220(16)
b (Å)	9.2166(10)	9.1488(11)	9.0831(11)	9.0019(13)	8.9874(13)	8.9082(12)
c (Å)	12.111(2)	12.024(2)	11.954(2)	11.875(2)	11.847(2)	11.8058(10)
Unit cell angles						
α (°)	90	90	90	90	90	90
β (°)	91.33(4)	91.57(4)	91.89(3)	92.23(4)	92.40(4)	92.80(3)
γ (°)	90	90	90	90	90	90
Volume (Å^3)	1399.7(10)	1367.0(11)	1339.5(10)	1313.2(11)	1301.8(13)	1283.7(16)
Z/*Z*'	4/1	4/1	4/1	4/1	4/1	4/1
Molecular volume (V/*Z*)	349.925	341.75	334.875	328.3	325.45	321.925
Calculated density (g/cm^3)	3.061	3.134	3.198	3.262	3.291	3.338
Absorption (mm^{-1})	23.444	24.004	24.496	24.987	25.205	25.562
F(000)	1152	1152	1152	1152	1152	1152
Crystal size (mm)	0.20 × 0.20 × 0.04	0.20 × 0.20 × 0.04	0.20 × 0.20 × 0.04	0.20 × 0.20 × 0.04	0.20 × 0.20 × 0.04	0.20 × 0.20 × 0.04
θ-range for data collection (°)	4.3690 to 23.5160	4.4270 to 23.8610	4.4570 to 22.3920	4.4920 to 22.3610	4.5000 to 22.6910	4.4050 to 23.3290
Min/max indices: *h*, *k*, *l*	-8 ≤ h ≤ 8, -11 ≤ k ≤ 11, -14 ≤ l ≤ 14	-7 ≤ h ≤ 7, -11 ≤ k ≤ 11, -14 ≤ l ≤ 14	-7 ≤ h ≤ 7, -11 ≤ k ≤ 11, -14 ≤ l ≤ 14	-7 ≤ h ≤ 7, -10 ≤ k ≤ 10, -14 ≤ l ≤ 14	-7 ≤ h ≤ 7, -10 ≤ k ≤ 10, -14 ≤ l ≤ 14	-4 ≤ h ≤ 4, -11 ≤ k ≤ 11, -14 ≤ l ≤ 14
Reflect. Collected/unique	6801/968	6748/966	6584/969	6047/913	6372/942	6033/820
Data/restrains/parameters	968/118/120	966/60/120	969/114/121	913/105/121	942/132/121	820/136/120
Goodness-of-fit on F^2	1.081	1.082	1.114	1.043	1.045	1.100
Final R_1/wR_2 (I>2σ1)	0.0647/0.1448	0.0594/0.1254	0.0654/0.1415	0.0821/0.2044	0.0665/0.1526	0.0791/0.1707
R_1/wR_2 (all data)	0.1137/0.1695	0.1038/0.1448	0.1189/0.1749	0.1343/0.2529	0.1146/0.1875	0.1091/0.1929
Largest diff. peak/hole (e.Å^{-3})	0.86/-1.44	1.11/-1.05	1.61/-1.50	1.23/-2.64	0.87/-2.30	1.67/-1.33

$w=1/(\sigma^2F_o^2+w_1^2*P^2+w_2*P)$, where $P=(Max(F_o^2,0)+2*Fc^2)$

Table S5. Detailed crystallographic data for ACE_2PbBr_4 at high-pressure (2.3-4.01 GPa) and RT.

Pressure	**2.30 GPa**	**2.69 GPa**	**2.94 GPa**	**3.36 GPa**	**3.65 GPa**	**4.01 GPa**
	phase II	phase II	phase II	phase II	phase II	phase II
CCDC numbers	2453056	2369887	2369888	2369889	2369890	2369891
Crystal system	monoclinic	monoclinic	monoclinic	monoclinic	monoclinic	monoclinic
Space group	$P2_1/c$	$P2_1/c$	$P2_1/c$	$P2_1/c$	$P2_1/c$	$P2_1/c$
Unit cell dimensions						
a (Å)	12.21(3)	12.168(2)	12.158(8)	12.181(18)	12.102(13)	12.069(12)
b (Å)	8.8936(9)	8.828(5)	8.7360(9)	8.6179(17)	8.6032(12)	8.5456(14)
c (Å)	11.7447 (18)	11.734(9)	11.7213(17)	11.680(4)	11.653(3)	11.620(3)
Unit cell angles						
α (°)	90	90	90	90	90	90
β (°)	93.00(5)	92.99(3)	93.43(3)	93.59(7)	94.33(5)	94.39(5)
γ (°)	90	90	90	90	90	90
Volume ($Å^3$)	1273(3)	1258.8(13)	1242.7(8)	1223.7(19)	1209.8(13)	1194.9(13)
Z/Z'	4/1	4/1	4/1	4/1	4/1	4/1
Molecular volume (V/*Z*)	318.25	314.7	310.675	305.925	302.45	298.725
Calculated density (g/cm^3)	3.365	3.404	3.448	3.501	3.541	3.585
Absorption (mm^{-1})	25.771	26.067	26.404	26.815	27.123	27.460
F(000)	1152	1152	1152	1152	1152	1152
Crystal size (mm)	0.20 x 0.20 x 0.04	0.22 x 0.16 x 0.04	0.19 x 0.15 x 0.04	0.19 x 0.15 x 0.04	0.19 x 0.15 x 0.04	0.19 x 0.15 x 0.04
θ-range for data collection (°)	3.4330 to 27.1090	4.9960 to 25.0360	3.4280 to 23.6730	3.4120 to 26.2160	3.4660 to 24.0470	3.4890 to 23.3110
Min/max indices: *h*, *k*, *l*	-3 ≤ h ≤ 3, -11 ≤ k ≤ 11, -15 ≤ l ≤ 15	-15 ≤ h ≤ 15, -8 ≤ k ≤ 8, -11 ≤ l ≤ 10	-8 ≤ h ≤ 8, -10 ≤ k ≤ 11, -14 ≤ l ≤ 14	-8 ≤ h ≤ 8, -10 ≤ k ≤ 11, -14 ≤ l ≤ 13	-8 ≤ h ≤ 8, -10 ≤ k ≤ 11, -14 ≤ l ≤ 14	-7 ≤ h ≤ 8, -10 ≤ k ≤ 10, -14 ≤ l ≤ 14
Reflect. Collected/unique	5487/695	5595/991	5906/969	3299/886	4190/916	3305/805
Data/restrains/parameters	695/105/121	991/50/120	969/116121	886/136/121	916/137/121	805/130/120
Goodness-of-fit on F^2	1.087	0.996	0.998	0.990	0.993	1.049
Final R_1/wR_2 (I>2σ1)	0.0832/0.2282	0.1000/0.2093	0.0772/0.1845	0.0962/0.2173	0.0925/0.2196	0.0822/0.1817
R_1/wR_2 (all data)	0.1114/0.2591	0.2291/0.2856	0.15850.2443	0.1879/0.2912	0.1831/0.2925	0.1665/0.2517
Largest diff. peak/hole (e.$Å^{-3}$)	1.20/-1.07	1.28/-1.19	2.00/-1.60	2.10/-2.04	2.08/-2.20	2.03/-1.45

$w=1/(\sigma^2F_o^2+w_1^2*P^2+w_2*P)$, where $P=(Max(F_o^2,0)+2*Fc^2)$

Table S6. Detailed crystallographic data for ACE_2PbBr_4 at high-pressure (4.2-6.8 GPa) and RT.

Pressure (GPa)	**4.20**	**4.65***	**5.20**	**5.70**	**6.20**	**6.80 GPa**
	phase II	phase II	phase III	phase III	phase IV	phase V
CCDC numbers	2369892		2453057	2453055	2453053	2453054
Crystal system	monoclinic	monoclinic	monoclinic	monoclinic	monoclinic	triclinic
Space group	$P2_1/c$	$P2_1/c$	$P2_1/c$	$P2_1/c$	$P2_1/c$	$P\bar{1}$
Unit cell dimensions						
a (Å)	12.05(3)	11.96(9)	11.947(7)	11.859(6)	11.499(5)	11.438(4)
b (Å)	8.535(4)	8.52(1)	8.4621(4)	8.4242(3)	8.376(4)	8.441(6)
c (Å)	11.603(5)	11.59(3)	34.5859(11)	34.4721(10)	11.84(5)	11.4999(15)
Unit cell angles						
α (°)	90	90	90	90	90	90.58(3)
β (°)	94.69(12)	94.7(4)	94.414(11)	94.556(11)	94.15(12)	94.296(18)
γ (°)	90	90	90	90	90	90.48(4)
Volume (Å^3)	1189(3)	1177(9)	3486.1(19)	3432.9(19)	1137(5)	1107.1(9)
Z/*Z*'	4/1	4/1	12/3	12/3	4/1	4/2
Molecular volume (V/*Z*)	297.25	294.323	290.508	286.075	284.25	276.775
Calculated density (g/cm^3)	3.602	-	3.687	3.736	3.767	3.870
Absorption (mm^{-1})	27.588	-	28.237	28.675	28.849	29.638
F(000)	1152	-	3456	3456	1152	1152
Crystal size (mm)	0.19 x 0.15 x 0.04	--	0.19 x 0.15 x 0.03	0.19 x 0.15 x 0.03	0.20 x 0.20 x 0.04	0.20 x 0.20 x 0.03
θ-range for data collection (°)	3.4890 to 23.4460	-	2.3500 to 30.3380	2.3520 to 27.5460	1.9390 to 22.2510	1.7720 to 25.8290
Min/max indices: *h*, *k*, *l*	-7 ≤ h ≤ 7, -10 ≤ k ≤ 9, -13 ≤ l ≤ 14	-	-4 ≤ h ≤ 4, -10 ≤ k ≤ 10, -43 ≤ l ≤ 43	-3 ≤ h ≤ 3, -10 ≤ k ≤ 11, -45 ≤ l ≤ 45	-6 ≤ h ≤ 5, -9 ≤ k ≤ 10, -13 ≤ l ≤ 13	-13 ≤ h ≤ 10, -6 ≤ k ≤ 6, -14 ≤ l ≤ 14
Reflect. Collected/unique	1898/666	-	10606/1916	18927/1986	1769/553	3137/879
Data/restrains/parameters	666/134/120	-	1916/608/358	1986/486/358	553/107/97	879/322/239
Goodness-of-fit on F^2	1.011	-	1.063	1.055	1.097	1.182
Final R_1/wR_2 (I>2σ1)	0.0887/0.1898	--	0.1261/0.3269	0.1228/0.3218	0.1377/0.3250	0.1085/0.2628
R_1/wR_2 (all data)	0.2021/0.2787	-	0.1476/0.3470	0.1562/0.3550	0.2152/0.3908	0.1490/0.3122
Largest diff. peak/hole (e.Å^{-3})	1.18/-1.15	-	2.26/-1.32	1.84/-1.25	0.77/-0.78	0.88/-1.06

*$w=1/(\sigma^2F_o^2+w_1^2*P^2+w_2*P)$, where $P=(Max(F_o^2,0)+2*Fc^2)$; *cf. Experimental Section in the Article.*

a. Calculation of the distortion parameters Δ and σ

The distortion of the $[PbBr6]_4^-$ octahedra were calculated accordingly to the previously reported methods.[3-5] For the calculation of the octahedra deformation (Δ*d*) the following equation was applied:

$$\Delta d \ = \frac{1}{6}\sum\left[\frac{d_n - d}{d}\right]^2,$$

where d_n are the six individual Pb-Br bond distances and *d* is the mean Pb-Br length.

The bond angles variance (σ^2) were estimated with equation:

$$\sigma^2 = \sum_{n=12}^{i=1}(\theta i - 90)^2/11,$$

where θ_i is the individual Br-Pb-Br angle.

Table S7. The bond length distortions of the individual PbB_{r6} octahedra at different pressures for isostructural phases I and II.

	phase I	phase I	phase I	phase I	phase I	phase II	phase II	phase II	phase II	phase II	phase II	phase II	phase II
Pressure (GPa)	0.50	0.85	1.20	1.55	1.89	2.10	2.30	2.69	2.94	3.36	3.65	4.01	4.20
Pb1-Br1	2.918	2.912	2.902	2.898	2.884	2.884	2.858	2.886	2.879	2.888	2.891	2.879	2.885
Pb1-Br2	3.104	3.083	3.060	3.038	3.028	3.037	2.996	3.010	2.982	2.982	2.964	2.945	2.965
Pb1-Br2'	3.166	3.135	3.114	3.096	3.102	3.077	3.061	3.068	3.063	3.042	3.045	3.041	3.004
Pb1-Br3	2.929	2.928	2.928	2.929	2.924	2.927	2.922	2.930	2.924	2.910	2.935	2.930	2.917
Pb1-Br4	2.929	2.932	2.928	2.914	2.916	2.912	2.920	2.887	2.898	2.883	2.888	2.889	2.879
Pb1-Br4"	3.211	3.168	3.144	3.128	3.118	3.102	3.119	3.096	3.092	3.092	3.085	3.073	3.080
**d**	**3.043**	**3.026**	**3.013**	**3.001**	**2.995**	**2.990**	**2.979**	**2.980**	**2.973**	**2.966**	**2.968**	**2.960**	**2.955**
**Δd**	**0.01478**	**0.01112**	**0.00939**	**0.00831**	**0.00854**	**0.00727**	**0.00800**	**0.00701**	**0.00653**	**0.00634**	**0.00551**	**0.00534**	**0.00504**

i=-x,0.5+y,0.5-z; ii=x,0.5-y,-0.5+z;

Table S8. The bond length distortions of the individual $PbBr_6$ octahedra at different pressures for phases III – V, the indices A, B and C labeling independent octahedra.

	phase III	phase III
Pressure (GPa)	5.2	5.7
Pb1A-Br1A	3.003	2.988
Pb1A-Br2A	2.970	2.953
Pb1A-Br3A	2.856	2.819
Pb1A-Br4A	2.954	2.944
Pb1A-Br4B	2.920	2.907
Pb1A-Br2C	3.002	2.966
**d_A**	**2.951**	**2.930**
**Δd_A**	**0.00261**	**0.00304**
Pb1B-Br1B	2.864	2.853
Pb1B-Br2B	2.941	2.956
Pb1B-Br2B'	3.082	3.031
Pb1B-Br3B	2.842	2.832
Pb1B-Br4B	2.904	2.882
**d_B**	**2.939**	**2.923**
**Δd_B**	**0.00810**	**0.00769**
Pb1B-Br4C''	3.001	2.986
Pb1C-Br1C	2.892	2.850
Pb1C-Br2C'''	2.975	2.974
Pb1C-Br3C	3.024	3.049
Pb1C-Br4C	2.822	2.788
Pb1C-Br2A	2.984	2.963
Pb1C-Br4A^{iv}	3.085	3.078
**d_C**	**2.964**	**2.950**
**Δd_C**	**0.00735**	**0.01053**
**$\bar{d}$**	**2.951**	**2.934**
**$\Delta\bar{d}$**	**0.00538**	**0.00607**
i=1-x,0.5+y,0.5-z; ii=1-x,-1/2+y,1/2-z; iii=x,1+y,z; iv=1-x,1-y,1-z		

	phase IV
Pressure (GPa)	6.2
Pb1-Br1	2.852
Pb1-Br2	2.839
Pb1-Br2'	3.153
Pb1-Br3	2.804
Pb1-Br4	2.919
Pb1-Br4''	3.109
**d**	**2.946**
**Δd**	**0.01843**
i=1-x,0.5+y,0.5-z; ii=x,0.5-y,-0.5+z;	

	phase V
Pressure (GPa)	6.8
Pb1A-Br1A	2.955
Pb1A-Br2A	2.840
Pb1A-Br3A	2.662
Pb1A-Br4A	2.943
Pb1A-Br2C	3.073
Pb1A-Br4B	2.907
**d_A**	**2.897**
**Δd_A**	**0.01584**
Pb1B-Br1B	2.839
Pb1B-Br2B	2.875
Pb1B-Br4B	3.089
Pb1B-Br1A'	2.942
Pb1B-Br3A''	3.234
Pb1B-Br4A'''	2.720
**d_B**	**2.950**
**Δd_B**	**0.02848**
**$\bar{d}$**	**2.923**
**$\Delta\bar{d}$**	**0.02287**
i=1-x,-y,-z; ii=1-x,1-y,-z; iii=x,y,-1+z	

Table S9. Bond angle variance of the individual $PbBr_6$ octahedra in different pressures.

	phase I	phase I	phase I	phase I	phase I	phase II	phase II	phase II	phase II	phase II	phase II	phase II	phase II
	0.50	0.85	1.20	1.65	1.89	2.10	2.30	2.69	2.94	3.36	3.65	4.01	4.2
Br1-Pb1-Br3	89.27	89.25	89.42	89.33	90.01	89.82	90.83	90.17	90.14	90.19	90.65	91.53	92.13
Br1-Pb1-Br4	85.82	85.02	84.36	83.39	82.85	82.66	82.31	82.05	81.24	80.34	80.13	79.52	79.76
Br1-Pb1-Br2	85.90	85.58	85.09	84.56	83.96	84.43	82.41	83.92	83.35	84.02	83.30	82.57	82.20
Br1-Pb1-Br4''	80.56	80.22	79.80	79.63	79.44	79.07	78.77	78.72	78.31	78.27	77.43	77.26	78.05
Br2-Pb1-Br2'	96.24	96.64	96.95	97.17	97.37	96.82	98.51	97.17	97.09	96.39	97.01	97.17	98.59
Br3-Pb1-Br2'	88.56	88.48	88.45	88.80	88.52	88.76	88.09	88.74	89.29	89.41	89.13	88.94	87.26
Br4''-Pb1-Br2'	101.20	102.88	104.53	106.49	107.67	109.25	110.43	111.08	112.84	114.41	116.12	117.31	117.57
Br4-Pb1-Br2'	92.18	91.63	91.07	90.28	89.88	88.88	88.37	88.05	87.57	86.95	86.31	85.91	84.58
Br4''-Pb1-Br2	93.22	93.01	92.78	92.29	92.07	91.71	91.11	90.61	90.50	89.70	89.54	88.94	88.20
Br4-Pb1-Br2	91.98	92.05	92.05	92.13	92.21	92.12	92.33	92.68	92.48	92.96	92.92	93.28	93.29
Br4-Pb1-Br3	87.16	86.72	86.49	86.31	86.27	86.41	86.38	86.70	86.53	86.71	87.10	87.21	87.08
Br4''-Pb1-Br3	86.47	86.86	87.13	87.45	87.55	87.90	87.91	88.01	88.15	88.46	88.07	88.18	89.22
**σ^2**	**27.496**	**32.448**	**37.979**	**44.668**	**49.746**	**54.082**	**63.649**	**62.584**	**71.487**	**77.924**	**88.948**	**97.313**	**100.584**
Pb1-Br2-Pb1'''	165.59	164.46	163.25	161.42	160.50	159.72	158.09	157.77	156.25	154.92	153.70	152.55	151.96
Pb1-Br4-$Pb1^{iv}$	161.95	161.16	160.47	159.74	159.28	159.50	158.93	159.32	158.38	158.21	157.51	157.19	158.49

i=-x,0.5+y,0.5-z; ii=x,0.5-y,-0.5+z; iii= -x,-0.5+y,0.5-z; iv= x,0.5-y,0.5+z

Table S10. Bond angle variance of the individual $PbBr_6$ octahedra in different pressures, the indices A, B and C labeling independent octahedra.

	phase III	phase III
	5.2	5.7
Br1A-Pb1A-Br3A	103.67	104.65
Br1A-Pb1A-Br4A	86.71	86.80
Br1A-Pb1A-Br2C	74.23	73.35
Br1A-Pb1A-Br4B	95.23	96.54
Br2A-Pb1A-Br2C	97.60	98.22
Br2A-Pb1A-Br3A	85.11	84.36
Br2A-Pb1A-Br4A	96.67	96.33
Br2A-Pb1A-Br4B	83.81	81.95
Br3A-Pb1A-Br4A	80.12	80.01
Br3A-Pb1A-Br4B	88.00	89.17
Br4A-Pb1A-Br2C	87.05	86.72
Br4B-Pb1A-Br2C	104.81	104.11
**σ^2_A**	80.656	88.287
Br1B-Pb1B-Br2B'	94.36	93.61
Br1B-Pb1B-Br3B	84.25	84.45
Br1B-Pb1B-Br4B	89.50	90.57
Br1B-Pb1B-Br4C''	82.02	82.86
Br2B-Pb1B-Br2B'	95.48	95.75
Br4B-Pb1B-Br2B'	81.79	79.87
Br4C''-Pb1B-Br2B'	119.98	120.14
Br2B-Pb1B-Br3B	86.63	86.97
Br2B-Pb1B-Br4B	92.60	91.82
Br2B-Pb1B-Br4C''	92.43	91.76
Br3B-Pb1B-Br4B	80.04	80.79
Br3B-Pb1B-Br4C''	77.89	78.91
**σ^2_B**	115.176	113.553
Br1C-Pb1C-Br3C	87.09	86.87
Br1C-Pb1C-Br4C	83.36	84.48
Br1C-Pb1C-Br2A	95.26	94.85
Br1C-Pb1C-Br4A^{v}	92.83	92.69
Br2C'''-Pb1C-Br3C	83.64	84.41
Br2C'''-Pb1C-Br4C	84.22	83.25
Br2C'''-Pb1C-Br2A	91.16	90.99
Br2C'''-Pb1C-Br4A^{v}	95.50	95.76
Br3C-Pb1C-Br4C	81.02	81.78
Br3C-Pb1C-Br4A^{v}	75.06	74.83

	phase IV
	6.2
Br1-Pb1-Br3	96.01
Br1-Pb1-Br4	81.60
Br1-Pb1-Br2	78.91
Br1-Pb1-Br4''	81.22
Br2-Pb1-Br2'	99.76
Br3-Pb1-Br2'	87.06
Br4''-Pb1-Br2'	119.04
Br4-Pb1-Br2'	78.27
Br4''-Pb1-Br2	81.74
Br4-Pb1-Br2	97.36
Br4-Pb1-Br3	87.59
Br4''-Pb1-Br3	91.73
**σ^2**	**126.898**
Pb1-Br2-Pb1'''	143.91
Pb1-Br4-Pb1^{v}	164.24

	phase V
	6.8
Br1A-Pb1A-Br3A	86.13
Br1A-Pb1A-Br4A	100.44
Br1A-Pb1A-Br4B	83.21
Br1A-Pb1A-Br2C	75.20
Br2A-Pb1A-Br3A	85.12
Br2A-Pb1A-Br4A	94.36
Br2A-Pb1A-Br4B	80.55
Br2A-Pb1A-Br2C	114.43
Br3A-Pb1A-Br4A	81.24
Br3A-Pb1A-Br4B	90.60
Br4A-Pb1A-Br2C	96.25
Br4B-Pb1A-Br2C	92.89
**σ^2_A**	102.853
Br1B-Pb1B-Br2B	82.02
Br1B-Pb1B-Br4B	88.59
Br1B-Pb1B-Br3A''	90.37
Br1B-Pb1B-Br4A'''	79.64
Br2B-Pb1B-Br4B	75.61
Br2B-Pb1B-Br1A'	82.39
Br2B-Pb1B-Br4A'''	87.38
Br4B-Pb1B-Br1A'	98.47
Br4B-Pb1B-Br3A''	113.39
Br1A'-Pb1B-Br3A''	103.04
Br1A'-Pb1B-Br4A'''	88.36
Br3A''-Pb1B-Br4A'''	82.36
**σ^2_B**	108.606
**$\bar{\sigma}^2$**	**105.729**
Pb1A-Br3A-Pb1B''	147.42
Pb1A-Br1A-Pb1B'	163.37
Pb1A-Br4A-Pb1B^{v}	162.28
Pb1A-Br4B-Pb1B	169.53

Br4C-Pb1C-Br2A	85.54	84.55						
Br2A-Pb1C-Br4A^{iv}	118.49	118.88						
**σ^2_C**	110.759	111.954						
**$\bar{\sigma}^2$**	**102.197**	**104.598**						
Pb1A-Br4A-Pb1C	171.32	171.47						
Pb1A-Br2C-Pb1C	171.62	172.63						
Pb1A-Br4B-Pb1B	161.19	163.90						
Pb1B-Br2B-Pb1B'	152.19	152.90						
Pb1A-Br2A-Pb1C	141.73	141.44						
Pb1B'-Br4C-Pb1C	148.99	150.81						
i=1-x,0.5+y,0.5-z; ii=1-x,-0.5+y,0.5-z; iii=x,1+y,z; iv=1-x,1-y,1-z				i=1-x,0.5+y,0.5-z; ii=x,0.5-y,-0.5+z; iii=1-x,-0.5+y,0.5-z; iv= x,0.5-y,0.5+z			i=1-x,-y,-z; ii=1-x,1-y,-z; iii=x,y,-1+z; iv=x,y,1+z	

4. Raman Studies

Table S11. Wavenumber intercepts at zero pressure (w_0) and pressure coefficients (a=dw/dP), obtained from fitting of the experimental data by linear functions, for the two phases of ACE_2PbBr_4. Modes corresponding to MHy^+ are denoted in red color.

ambient pressure phase I		high-pressure phase II		high-pressure phase III		high-pressure phase IV		high-pressure phase V		Assignment*
ω_0 (cm^{-1})	α ($cm^{-1}GPa^{-1}$)	ω_0 (cm^{-1})	α ($cm^{-1}GPa^{-1}$)	ω_0 (cm^{-1})	α ($cm^{-1}GPa^{-1}$)	ω_0 (cm^{-1})	α ($cm^{-1}GPa^{-1}$)	ω_0 (cm^{-1})	α ($cm^{-1}GPa^{-1}$)	
		3319.3	3.04					3326.5	-0.01	$\nu(NH_2)$
3309.74	-0.31	3305.7	-1.57	3296.8	0.84	3311.5	-1.73	3307.4	-1.24	$\nu(NH_2)$
3251.9	0.28									$\nu(NH_2)$
								3199.6	3.25	$\nu(NH_2)$
3207.7	1.22	3205.6	2.27	3234.7	-4.13	3243.4	-5.55	3255.2	-7.01	$\nu(NH_2)$
3165.2	-1.51	3163.0	0.32	3162.4	0.12	3156.4	1.19			$\nu(NH_2)$
				3158.7	-1.72	3146.7	0.43			$\nu(NH_2)$
								3001.4	4.58	$\nu_{as}(CH_3)$
						2906.7	8.18	2883.1	12.35	$\nu_s(CH_3)$
2925.2	3.68	2923.7	4.24	2937.2	1.31	2962.9	-3.01	2920.7	3.34	$\nu_s(CH_3)$
								1665.3	5.94	$\nu(CN)$
1681.2	1.26	1680.4	1.79	1682.8	1.24	1708.3	-2.71	1655.9	4.96	$\nu(CN)$
								1664.3	1.06	$\nu(CN)$
1647.1	1.82									$\delta(NH_2)$
1629.8	-1.52	1627.3	0.46	1628.0	0.39	1637.4	-1.73	1601.1	3.31	$\delta(NH_2)$
1611.7	1.19	1615.5	0.32	1641.9	-5.17	1622.7	-2.08	1596.7	1.79	$\delta(NH_2)$
1556.7	-2.34	1558.6	-0.12							$\delta(NH_2)$
				1536.3	0.49	1535.9	0.33	1541.1	-0.54	$\delta(CH_3)$
1522.5	2.70	1525.0	0.95	1524.3	1.04	1543.9	-2.39			$\delta(CH_3)$
1506.6	2.61	1509.0	0.83	1509.2	1.16	1507.1	1.39	1503.2	1.97	$\delta(CH_3)$
								1455.7	-1.19	$\rho(NH_2)$

				1446.7	-3.53	1399.5	4.38	1417.1	1.89	ρ(NH_2)
				1407.1	1.05	1407.2	0.95	1419.6	-1.06	ρ(NH_2)
1139.2	-0.37	1139.0	0.06	1111.8	7.49	1177.3	-3.43	1129.7	3.33	ν_s(NCN)
1118.1	2.77	1120.8	1.16	1116.6	1.70	1127.2	-0.12	1090.4	5.22	ν_s(NCN)
1078.5	0.51	1079.7	-0.32	1072.3	1.31	1065.2	2.51	1054.1	4.16	δ(CC) out-of-plane
		1062.6	-3.45	1047.4	-0.02	1049.4	-0.31	1017.2	4.66	δ(CC) out-of-plane
		1007.5	1.13	1013.9	0.12	1017.2	-0.26	926.3	13.08	ρ(CH_3)
								890.7	6.69	ν(CC)
						904.8	3.68	880.6	7.19	ν(CC)
				896.0	3.45	903.1	2.26	885.5	4.58	ν(CC)
884.7	5.77	887.5	4.23	894.0	3.13	884.4	4.59	866.2	7.28	ν(CC)
				892.7	2.33	890.8	2.64	881.1	4.05	ν(CC)
747.7	-0.82	752.1	-3.11	682.6	12.88	768.2	-2.49	740.1	1.52	ω(NH_2)
				720.6	-1.75	703.3	1.08	712.4	-0.07	ω(NH_2)
681.6	-1.07	676.9	1.15							ω(NH_2)
								578.5	12.88	ω(NH_2)
646.4	1.24	648.6	0.47			680.7	-4.43			ω(NH_2)
				663.8	0.19	601.8	5.64	611.4	4.42	ω(NH_2)
622.1	2.73	636.0	-4.51							ω(NH_2)
595.5	-0.38	598.0	-1.53	514.9	15.97	484.6	20.15	563.8	8.49	τ(NH_2)
		563.8	-2.05	567.7	-0.59	525.6	6.71	516.3	8.22	τ(NH_2)
								534.5	1.97	τ(NH_2)
554.2	-7.88	538.7	0.17	520.7	2.94	548.8	-1.93	495.0	6.00	τ(NH_2)
		526.5	1.79							τ(NH_2)
518.2	2.80	520.9	1.21	518.6	1.57	530.1	-0.39	509.5	2.62	δ(NCN)
517.8	-0.73	516.1	0.39							δ(NCN)
		516.5	-2.76	502.1	1.26	502.8	1.19	496.9	2.07	δ(NCN)
								498.8	-0.19	δ(NCN)

								443.2	3.15	δ(CC) in-plane
				437.9	3.51	444.9	2.19	455.6	0.44	δ(CC) in-plane
441.7	1.72	440.8	2.15	446.8	1.15	447.7	0.94	435.1	2.82	δ(CC) in-plane
140.7	17.10					121.9	14.28	129.8	13.25	Pb-Br stretch
						158.3	3.79	96.4	13.00	Pb-Br stretch
129.6	7.50	130.6	6.78	135.6	6.04	150.1	1.45	150.1	1.23	Pb-Br stretch
111.6	4.72	105.6	8.46	127.7	4.35	141.8	0.23			Pb-Br stretch
								121.6	4.19	Pb-Br stretch
		101.6	5.61	126.2	0.99	111.9	3.45	93.8	6.38	Pb-Br bend
		73.9	6.98	60.7	9.75	107.1	1.84	119.4	0.08	Pb-Br bend
						83.4	3.87	76.4	4.93	Pb-Br bend
						111.3	-1.88	94.4	0.85	Pb-Br bend
		63.6	5.06	66.2	4.80	105.5	-2.38	74.1	2.49	Pb-Br bend

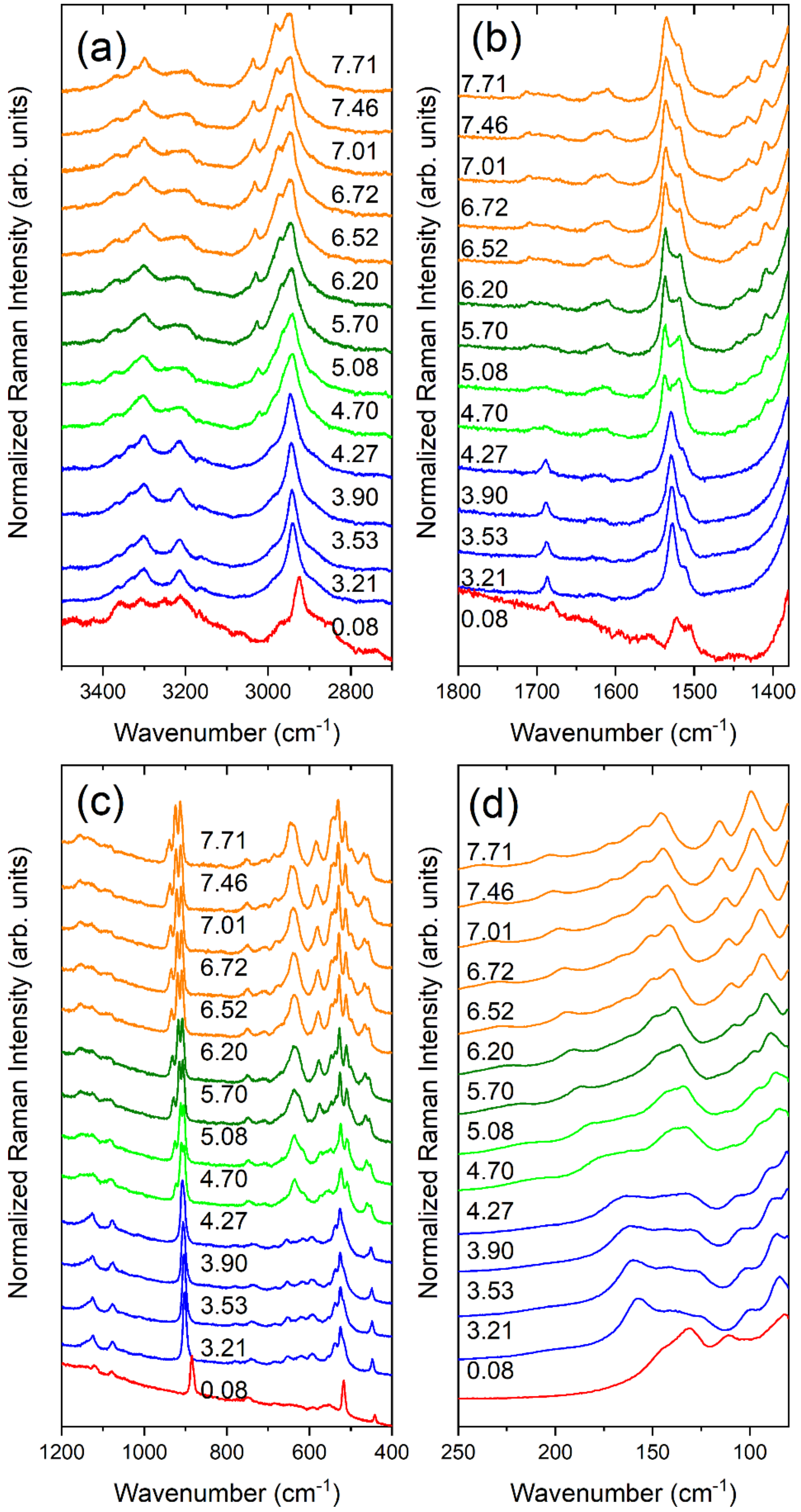

Figure S7. Raman spectra of ACE_2PbBr_4 under decompression in the (a) 3450-2750 cm^{-1}, (b) 1800-1380 cm^{-1}, (c) 1200-400 cm^{-1} and (d) 250-80 cm^{-1} range.

5. Optical Studies

The reflectance spectra are fitted with the formula given by equation (1)

$$R(E) = R_0 + R_A \mathrm{Re}\left(\frac{E_0 - E + i\Gamma}{(E_0 - E)^2 + \Gamma^2}\, e^{i\Theta}\right), \quad (1)$$

where R_A, E_0, and Γ is an amplitude, energy, and broadening of FE transition. R_0 is a background and Θ is a phase of this transition. Figure S8 shows some examples of the reflectance spectra fitted by equation 1. The part of the spectrum in the dashed box was not fitted, but it is worth noting here that in this spectral range another optical transition is possible at another point of the Brillouin zone. Moreover, above the fundamental excitonic transition we expect interband absorption, which also affects the reflectance spectrum. Therefore, the reflectance spectra are fitted only in the region of the fundamental FE transition.

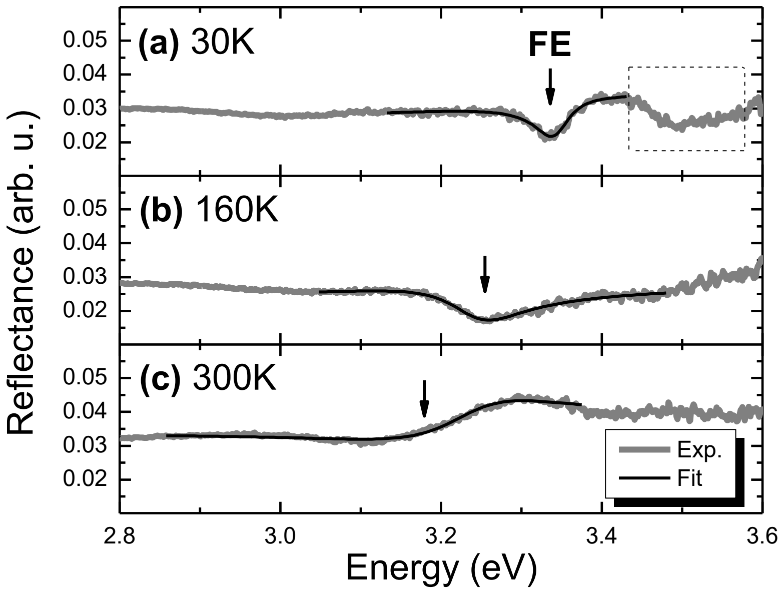

Figure S8. Examples of reflectance spectra measured at (a) 30 K, (b) 160 K, and (c) at 300 K fitted using equation (1).

The temperature dependence of the energy gap (and FE transition) in regular semiconductors can be fitted using the Bose–Einstein formula,[6] which can be written as equation (2):

$$\mathrm{E_0(T) = E_0(0)} - \frac{2\mathrm{a_B}}{\exp\left(\frac{\Theta_\mathrm{B}}{\mathrm{T}}\right) - 1}, \quad (2)$$

where a_B is the strength of the electron-average phonon interaction and Θ_B is the average phonon temperature. The same formula was used to fit the temperature dependence of the FE transition in ACE_2PbBr_4 observed in the reflectance spectrum, but reasonable parameters were not obtained. Therefore, the experimental data were finally fitted using a linear relationship with a coefficient of 0.65±0.05 meV/K. In general, the Bose-Einstein relation is non-linear in low temperature regime[6,7] and it is easy to see that this relation becomes linear for T >> Θ_B, i.e., $E(T) \approx E_0(0) - 2a_B \frac{T}{\Theta_B}$. From our fit of the experimental results with the Bose-Einstein formula we obtained that the Θ_B parameter is small. Therefore, linear fitting of FE transition is fully justified in this case. Furthermore, it is generally known that the temperature dependence of the band gap in perovskites behaves differently than in regular semiconductors.

The increase in the broadening of the FE transition with the temperature increase is caused by the interaction with acoustic (AC) and optical (LO) phonons according to equation (3)

$$\gamma(T) = \gamma_0 + \gamma_{AC}T + \frac{\gamma_{LO}}{\exp\left(\frac{\Theta_{LO}}{kT}\right)-1}, \quad (3)$$

where γ_0 represents the broadening invoked from temperature-independent mechanism, whereas the second term corresponds to the lifetime broadening due to electron-acoustical phonon interaction, where γ_{AC} is the acoustical phonon coupling constant, and the third term is related to the Fröhlich interaction with LO phonons. The γ_{LO} represents the strength of the exciton-LO phonon coupling, Θ_{LO} is the LO phonon energy and k is the Boltzmann constant. For FE transition in ACE_2PbBr_4 a good fit was obtained with the parameters: γ_0=21±4 meV, γ_{AC}=0.08±0.04 meV/K, γ_{LO}=190±40 meV, and Θ_{LO}=60±10 meV.

6. References

(1) Lertkiattrakul, M.; Evans, M. L.; Cliffe, M. J. PASCal Phyton: a Principal Axis Strain Calculator. *J. Open Source Software* **2023**, *8*, 5556.

(2) Cliffe, M. J.; Goodwin, A. L. PASCal: a Principal Axis Strain Calculator for Therml Expansion and Compressibility Determination. *J. Appl. Crystallogr.* **2012**, *45*, 1321−1329.

(3) Robinson, K.; Gibbs, G. V.; Ribbe, P. H. Quadratic Elongation: A Quantitative Measure of Distortion in Coordination Polyhedra. *Science* **1971**, *172*, 567−570.

(4) Gao, F. F.; Li, X.; Qin, Y.; Li, Z. G.; Guo, T. M.; Zhang, Z. Z.; Su, G. D.; Jiang, C.; Azeem, M.; Li, W.; Wu, X.; Bu, X. H. Dual-Stimuli-Responsive Photoluminescence of Enantiomeric Two-Dimensional Lead Halide Perovskites. *Adv. Opt. Mater.* **2021**, *3*, 2100003.

(5) Mao, L. Guo, P.; Kepenekian, M.; Hadar, I.; Katan, C.; Even, J.; Schaller, R. D.; Stoumpos, C. C.; Kanatzidis, M.G. Structural Diversity in White-Light-Emitting Hybrid Lead Bromide Perovskites. J. Am. Chem. Soc. 2018, 140, 13078−13088.

(6) Logothetidis, S.; Cardona, M.; Lautenschlager, P.; Garriga, M. Temperature Dependence of the Dielectric Function and the Interband Critical Points of CdSe. *Phys. Rev. B* **1986**, *34*, 2458.

(7) Lautenschlager, P.; Garriga, M.; Logothetidis, S.; Cardona, M. Interband Critical Points of GaAs and their Temperature Dependence. *Phys. Rev. B* **1987**, *35*, 9174.
